# Bridging the Gap to Bionic Motion: Challenges in Legged Robot Limb Unit Design, Modeling, and Control

**DOI:** 10.34133/cbsystems.0365

**Published:** 2025-08-19

**Authors:** Junhui Zhang, Jinyuan Liu, Huaizhi Zong, Pengyuan Ji, Lizhou Fang, Yong Li, Huayong Yang, Bing Xu

**Affiliations:** State Key Laboratory of Fluid Power and Mechatronic Systems, School of Mechanical Engineering, Zhejiang University, Hangzhou 310027, China.

## Abstract

Motivated by the agility of animal and human locomotion, highly dynamic bionic legged robots have been extensively applied across various domains. Legged robotics represents a multidisciplinary field that integrates manufacturing, materials science, electronics, and biology, and other disciplines. Among its core subsystems, the lower limbs are particularly critical, necessitating the integration of structural optimization, advanced modeling techniques, and sophisticated control strategies to fully exploit robots’ dynamic performance potential. This paper presents a comprehensive review of recent developments in the structural design of single-legged robots and systematically summarizes prevailing modeling approaches and control strategies. Key challenges and potential future directions are also discussed, serving as a reference for the future application of state-of-the-art manufacturing and control methodologies in legged robotic systems.

## Introduction

In recent years, robots have increasingly become integral in enhancing human life, particularly with the growing demand for mobile robots with high payload-to-weight ratios and dynamic capabilities [1–3]. However, most real-world environments are unstructured and harsh, consisting of granular substrates such as soft mud, quicksand, and gravel deposits. Traditional wheeled or tracked robots are prone to issues such as getting stuck or overturning in these terrains, hindering smooth and rapid movement and potentially causing damage to their structure, which have accelerated the development of legged robots, inspired by the way animals move in nature.

**Fig. 1.**
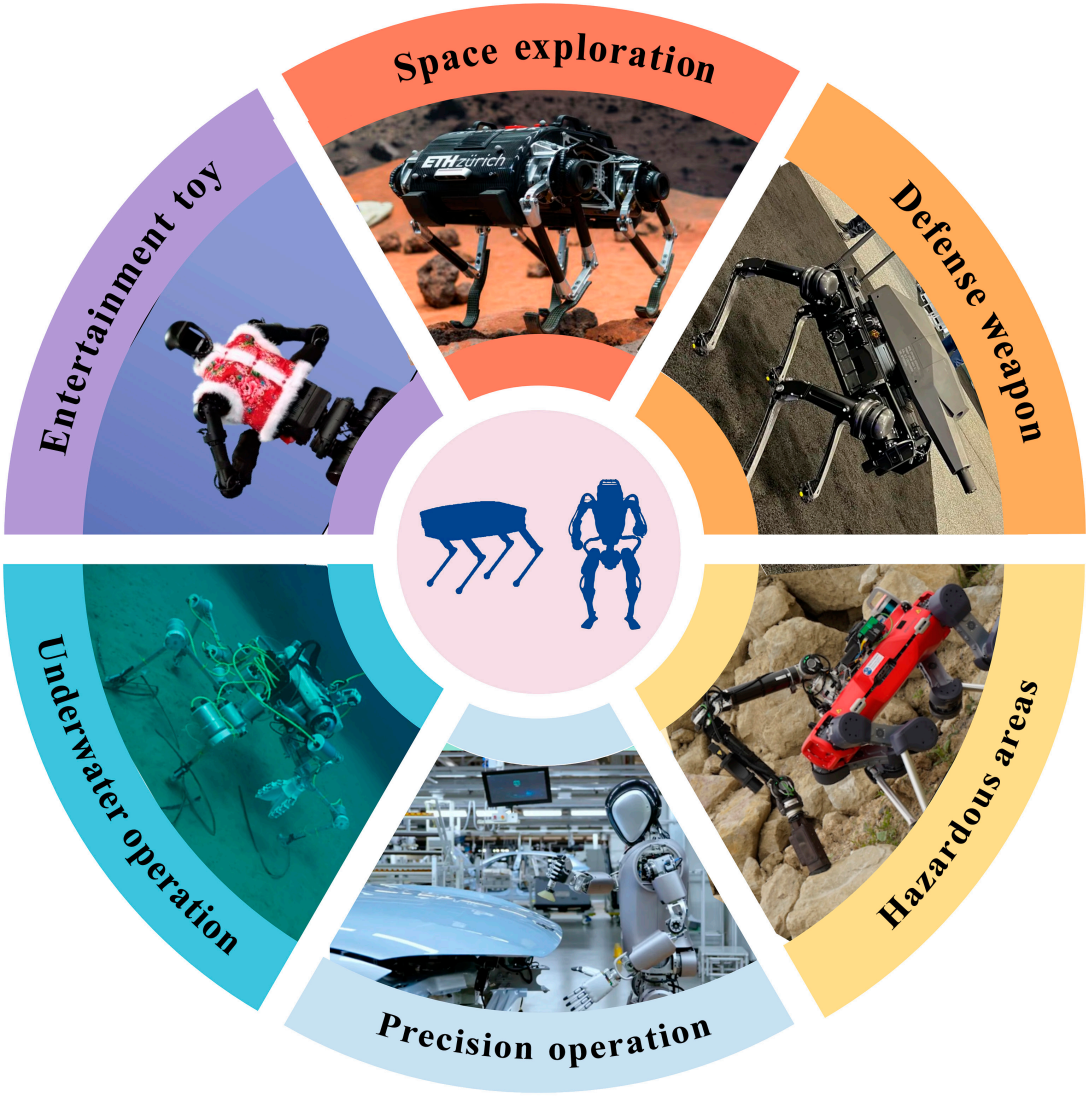
Application scenarios of legged robots in diverse environments.

Unlike traditional mobile robots, legged robots leverage their distinctive “leg” structures to traverse obstacles and adapt to uneven terrain, demonstrating exceptional mobility when confronted with pronounced undulations or soft ground [4,5], as shown in Table 1. Their excellent terrain adaptability and high flexibility enable them to perform tasks in complex and unstructured environments that are challenging for wheeled or tracked robots to accomplish [6,7], as shown in Fig. 1. Furthermore, the increasing attention on humanoid robots has substantially accelerated advancements in legged robotics, spurred by the demand for highly efficient and anthropomorphic locomotion capabilities.

**Table 1.** Comparative performance characteristics of mobile robot

Performance	Wheeled/Tracked robot	Legged robot
Moving speed	High	Medium
Stability	High	Medium
Maneuverability	Low	High
Terrain adaptability	Medium	High
Energy efficiency	High	Low
Control complexity	Low	High
Navigation over obstacles	Medium	High

However, the integration of limb-leg units into robots presents a range of intricate challenges within the research process. From the hardware manufacture perspective, leg structures are required to not only support the robot’s weight, but also generate sufficient actuation force to drive the entire system, including any additional payloads. An inherent trade-off exists between the imperative for structural light weighting to reduce gravitational loading [8,9] and the necessity of ensuring adequate load-bearing capacity and actuator output to meet task-specific dynamic performance demands [10,11]. Furthermore, the impact force generated when the foot lifts off or contacts the ground can affect the internal structure, thereby making impact mitigation a critical consideration in the overall design process [12]. From the control perspective, legged robots exhibit substantially greater kinematic and dynamic complexity compared to wheeled or tracked counterparts, owing to their increased degrees of freedom (DoFs) [13,14]. Besides, each leg must continuously transition between the swing phase and the support phase during locomotion, with varying speed, force, and torque requirement demands at each moment [15,16]. Such temporal variability imposes considerable challenges on the control architecture, necessitating high-precision, real-time coordination across multiple joints and actuators.

Due to the reasons mentioned above, the construction and analysis of complete legged robotic systems remain inherently complex and challenging. Compared to the complete multi-legged robots (MLRs), single-legged robots (SLRs) feature simpler configurations and typically admit a dynamic gait: hopping. Within each hopping cycle, the SLR can effectively emulate the motion behavior of each individual leg in MLRs, while dynamically interacting with the external environment. Consequently, SLRs serve as ideal research platforms, offering valuable theoretical insights and empirical data for addressing critical issues in structural design and dynamic control.

This article provides a comprehensive review of SLRs, focusing on their mechanical architectures, applications, as well as modeling and control strategies. The structure of the article is organized as follows: The “Hardware Structure of SLRs” section introduces the hardware development of SLRs and extends research to MLRs. The “Control Strategy for SLRs” section discusses the classification and progress of modeling and control strategies. The “Future Research and Challenges” section proposes future research directions and key challenges. Finally, the “Conclusions” section provides the concluding remarks.

## Hardware Structure of SLRs

The hardware structure directly influences SLRs’ mobility, stability, and task execution efficiency. On the one hand, the structure must balance rigidity and adaptability to prevent motion failure or trajectory deviation caused by vibrations or deformations during the stance phase. On the other hand, upon contact with the ground, the structures should possess cushioning capabilities to absorb impact forces, thereby preventing potential damage to the components. Based on the variations in leg joint configurations, SLRs can be categorized into 2 types: telescopic and articulated.

### Telescopic SLRs

**Fig. 2.**
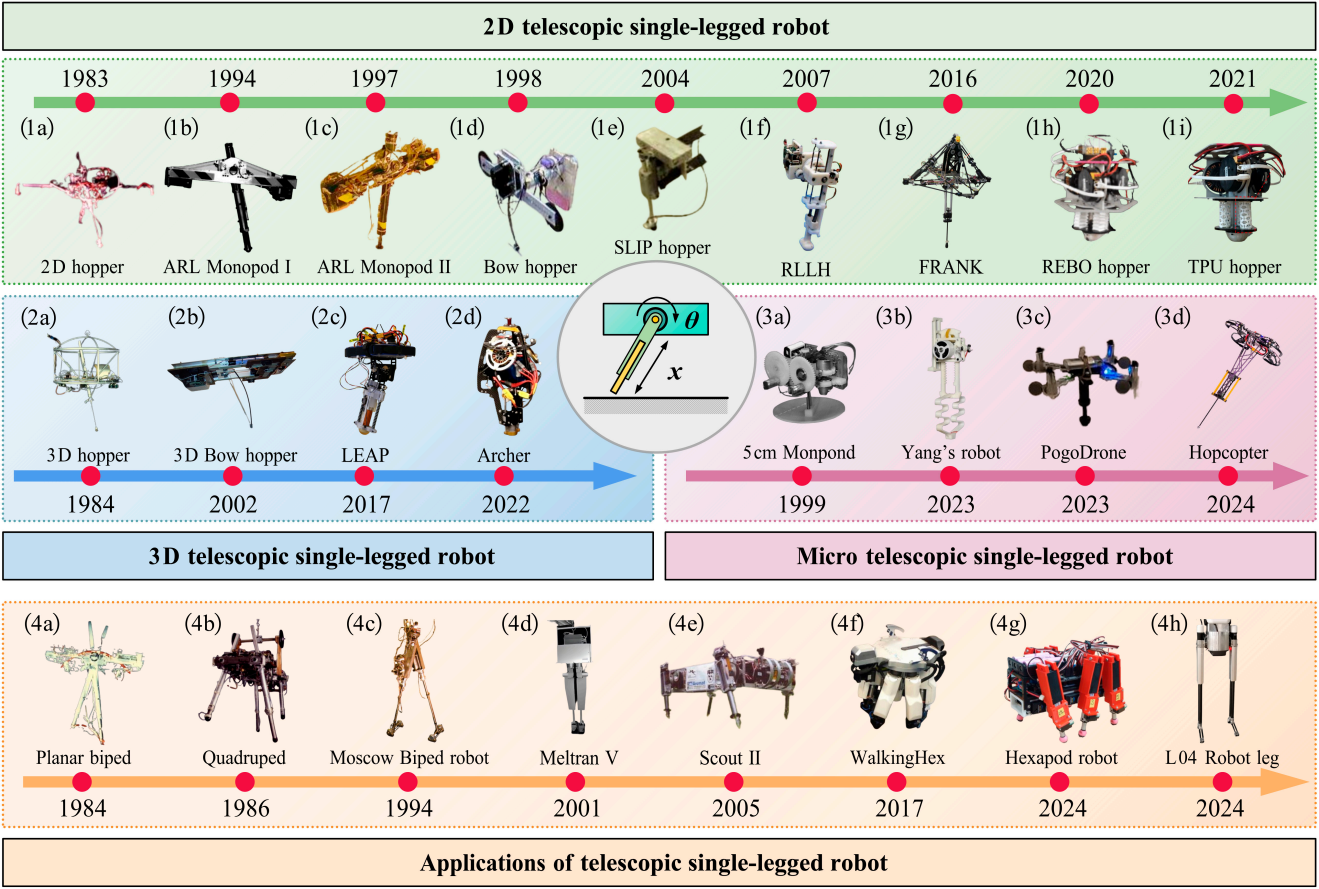
Categorization and application domains of telescopic SLRs .

Telescopic SLRs possess a vertical telescoping DoF in one direction, typically actuated via pneumatic cylinders, hydraulic actuators, or electric motors with linear or torsional springs. Their jumping capability is primarily derived from the extension and compression of springs or rods in contact with the ground. Owing to their simple mechanical design and relatively straightforward jumping planning methods, telescopic SLRs have been widely adopted in early studies. The classification of telescopic SLRs and their representative applications in MLRs are illustrated in Fig. 2.

#### Classification of telescopic SLRs

In this paper, based on the differences in the hip DoFs and mass of telescopic SLRs, the current research is divided into three: 2-dimensional (2D) telescopic SLRs and 3-dimensional (3D) telescopic SLRs (>200 g), as well as micro telescopic SLRs (<200 g) for special mission environments.

##### 2D telescopic SLRs

2D telescopic SLRs are characterized by a single rotational DoF at the hip joint, enabling planar jumping movements restricted to the sagittal plane (i.e., forward and backward directions only).

A representative early example is the robot developed in 1983 by Professor Raibert [17,18] [Fig. 2(1a)]. This prototype is powered by 2 pneumatic cylinders: one controls the rotation of the hip joint to control the pitch and direction of the robot, while the other adjusts the extension and compression to store and release energy. It is capable of vertical hopping in place, hopping at various forward speeds, and leaping over small obstacles. The success of Raibert’s hopper has markedly catalyzed a surge in research within the field of SLRs. Building upon the previous McGill Hoppe structure [19], Gregorio [20,21] developed the ARL Monopod I [Fig. 2(1b)] consisting of 2 components: a telescopic leg and a body, which are connected by an actuated hip joint. The robot achieves an average power consumption of approximately 125 W, thereby demonstrating the feasibility of electrically driven legged robots. In response to the substantial energy waste during the oscillation, Ahmadi and Buehler [22] further revised the hip structure by incorporating flexible components, resulting in the ARL Monopod II [Fig. 2(1c)]. Although the overall weight of the prototype increased, the average power has only a servo motor with a gear reducer to rotate the leg consumption, which was reduced to 48 W at a maximum dynamic hopping speed of 1.25 m/s, leading to a notable improvement in energy efficiency.

Influenced by the widely adopted model control approach, researchers identified that SLRs designed to closely resemble the spring-loaded inverted pendulum (SLIP) model would facilitate more precise control. Zeglin [23,24] developed the first elastic bow robot [Fig. 2(1d)], which aligns more closely with the SLIP model than spring-driven designs, addressing the issues such as low energy efficiency and landing vibrations. Subsequently, Sato [25] firstly proposed the SLIP Hopper, which strictly adheres to the simplified SLIP model shown in Fig. 2(1e). However, energy losses along the leg extension direction limit its capability for achieving sustained vertical hopping.

The aforementioned SLRs are typically evaluated on flat laboratory surfaces, whereas the uneven and rugged terrains in real-world environments pose entirely new challenges to their stability and adaptability. Sheikh [26] proposed a reconfigurable leg length hopper (RLLH) with passive compliance, as shown in Fig. 2(1f). By adjusting the rate and amplitude of leg length variation, the RLLH can effectively adapt to changes in coupling stiffness and damping across various terrains. Furthermore, the robot named FRANK was proposed to investigate the feedback control on uneven terrain [Fig. 2(1g)] [27]. FRANK contains a cable-driven series elastic actuator to inject energy into the system, a planar angle actuator to regulate the forward touchdown angle, and an additional actuator to keep the foot from slipping during the stance phase. By regulating the energy stored in the spring, precise adjustments to the jumping height can be achieved.

Conventional telescopic legged robots typically rely on elastic elements such as linear or torsional springs, which typically suffer from energy losses and limit overall efficiency. To overcome the limitations of soft materials in terms of energy density and responsiveness, structured mechanical metamaterials have emerged as a promising solution, which enhance the flexibility, energy efficiency, and operational stroke of robots [28,29].

Several robots have drawn inspiration from the properties of mechanical metamaterials to develop lightweight, flexible components. The hopper [Fig. 2(1h)], developed by Chen et al. [30], leverages mechanical metamaterials by utilizing the new reconfigurable expanding bistable origami (REBO) pattern as spring elements. The robot’s flexible leg is composed of 3 parallel REBO springs, each independently actuated via a dedicated motor, pulley, and tendon. By modulating the tendon lengths, the leg enables the robot to perform forward, backward, and vertical jumps with high agility and control. Building upon this foundation, Lee et al. [31] proposed a 3D-printed auxetic tubular spring with tunable stiffness and 35.2% higher energy storage than linear springs for SLRs. Integrated into the thermoplastic polyurethane (TPU) hopper [Fig. 2(1i)] via tendon-driven actuation, the spring enables stable 1-DoF and 2-DoF hopping, while simultaneously achieving a notable improvement in energy efficiency and compact force.

However, any lateral impact disturbance during legged locomotion will induce an unintended lateral velocity, causing the movement to deviate from its original direction. By incorporating the additional DoF, the 3D telescopic SLR can more accurately reflect real-world mission scenarios, allowing for better handling of lateral disturbances and more complex environments.

##### 3D telescopic SLRs

Compared to the 2D telescopic SLR, the hip joint of the 3D telescopic SLR not only offers freedom in the vertical direction but also allows for swinging parallel to the jumping direction. This added capability enables the robot to achieve hopping in all directions: forward, backward, left, and right.

Building upon the foundational design of 2D telescopic SLR, researchers expanded the structure to 3D to better accommodate complex locomotion. In 1984, Raibert et al. [32] further developed a 3D hopper [Fig. 2(2a)], consisting of 2 main parts: an aluminum body and a pneumatic jumping leg. The hip actuator was modified to use hydraulic drive, while the leg actuator still employed pneumatic drive. The robot achieves a forward speed of 2.2 m/s, a lateral speed of 1.8 m/s, and a maximum stride of 0.79 m in a laboratory setting. In a subsequent advancement, the 3D bow-legged SLR [Fig. 2(2b)] replaced the original hip hinge with a universal joint [33]. This modification allowed the robot not only to move forward and backward but also to achieve lateral displacement. This robot is primarily affected by air resistance, which increases the complexity of control.

With the proposal of various actuators, novel configurations have emerged for the 3D telescopic SLR design. In 2017, Batts et al. [34] applied voice coil actuators to propose a spatial jumping robot, driven by a linear elastic actuator in parallel (LEAP), aiming to better adapt to rugged and complex environments, as shown in Fig. 2(2c). This design partially offloads the weight onto the spring, thereby reducing the force and power requirements of the actuators. Additionally, Csomay-Shanklin et al. [35] utilized the flywheel for attitude adjustment control of the 3D robot “Archer” [Fig. 2(2d)]. Height modulation is realized through a cable mechanism controlling the compression and driving of the spring, while body posture is achieved through 3 flywheels inside the torso. These flywheels maintain the body in an upright position through differential rotation and adjust the foot position as needed, thus enabling the robot to achieve various functions.

##### Micro telescopic SLRs

Miniature robots present notable advantages, such as reduced manufacturing costs and improved energy efficiency. Furthermore, their small size and lightweight design enhance portability and enable integration with humans or other carrier platforms. These features make them highly adaptable for a wide range of specialized applications [36,37]. The telescopic leg configuration serves as a fundamental design approach, balancing size constraints with hopping performance. In this paper, robots with the telescopic configuration with a mass of less than 200 g are classified as micro telescopic SLRs.

In 2000, Wei et al. [38] constructed an autonomous SLR with a volume of less than 5 cm^3^ [Fig. 2(3a)], which is driven by rotating 2 discs attached to the leg. It could travel at a speed of 7.75 cm/s. However, there are 2 main drawbacks: the inability to actively control its direction due to limitations in its DoFs, and the vibration caused by eccentric mass, leading to unnecessary energy consumption. Yang et al. [39] proposed a compact robot [Fig. 2(3b)], featuring a rectangular rack and active clutch to ensure precise timing and efficient linear motion. The top-mounted parallel spring functions to minimize energy loss and augment propulsion during takeoff, whereas the tip-mounted series spring converts landing impact into reusable energy.

To further improve the jumping capabilities of robots under strict size constraints, some engineers have drawn inspiration from insects such as fleas and grasshoppers [40,41], which leverage their lightweight anatomical structures to achieve rapid locomotion by combining jumping with brief aerial phases. One notable example is the PogoDrone [Fig. 2(3c)], a jumping–flying hybrid robot that integrates a quadrotor with a passive jumping mechanism [42]. The incorporation of passive jumping markedly reduces the power required for locomotion, thereby enhancing energy efficiency and extending maximum flight duration. Similarly, Bai et al. [43] proposed the robot Hopcopter [Fig. 2(3d)], which integrates a micro quadcopter with a passive elastic telescopic leg. By utilizing propeller thrust during the flight phase, the robot adjusts jump height and direction, enabling midair burst jumps that enhance instantaneous acceleration and maneuverability. These approaches demonstrate the transformative potential of integrating flight mechanics into micro SLRs.

Scale effects enable certain performance at small physical dimensions that are unattainable at larger scales [44]. For example, froghoppers exert forces of more than 400 body weight, a capability that is physiologically unattainable for larger animals [45]. Through Froude number analysis [46], it is evident that smaller robots are inherently better suited for jumping gaits, echoing the locomotion strategies seen in small animals. With decreasing physical dimensions of robots, the moments of inertia are substantially reduced, allowing for higher accelerations and greater force-to-mass ratios. Consequently, micro telescopic SLRs can achieve higher jump heights and longer distances. Besides, their compact design not only enhances agility and task efficiency but also mitigates the risk of mechanical damage during high-impact operations. However, miniaturization also introduces new challenges: air resistance becomes non-negligible, and control systems must handle faster dynamics and greater sensitivity to disturbances.

#### Applications of telescopic SLRs

Telescopic structural legs are favored for their simple and convenient configuration, as well as their compact working space during motion. Table 2 summarizes representative telescopic SLRs, their physical parameters and mobility. Consequently, some MLRs operating in less complex environments have adopted similar telescopic designs to improve mobility and adaptability [45]. The earliest MLRs to implement the telescopic configurations were the bipedal and quadrupedal robots proposed by Raibert et al. [47] in the mid-1980s [Fig. 2(4a and 4b)], capable of performing various gaits such as trotting, hopping, and diagonal running.

**Table 2.** Structural parameters of typical telescopic SLRs

Telescopic SLR	C	*D*/m	*M*/kg	Mobility	Actuator	Refs.
Raibert 2D hopper ^a^	2D	/	8.49	0.65 (JH)	P	[17,18]
ARL Monopod I	2D	/	15.00	0.20 (JH), 1.20 (Fv)	E	[20,21]
ARL Monopod II	2D	0.64 (l)	15.00	0.75 (JH), 1.25 (Fv)	E	[22]
Bow hopper	2D	0.25 (l)	4.00	0.50 (JH), 1.00 (Fv)	E	[23,24]
SLIP hopper	2D	0.12 (l)	0.54	0.17 (JH)	E	[25]
RLLH	2D	0.26×0.04×0.05 (L×W×H)	0.87	0.25 (JH)	E	[26]
FRANK	2D	0.54 (l)	8.15	0.80 (JH)	E	[27]
REBO hopper	2D	0.21 (W), 0.23 (H)	2.53	0.27 (JH), 0.20 (Fv)	E	[30]
TPU hopper	2D	0.21 (W), 0.23 (H)	2.50	0.06 (Fv)	E	[31]
Raibert 3D hopper ^a^	3D	0.76×1.10 (W×H)	17.00	0.58 (JH)	P/Hy	[32]
3D bow hopper	3D	0.45×0.29×0.05 (L×W×H), 0.23 (l)	2.00	0.50 (JH)	E	[33]
LEAP	3D	0.06 (l)	2.47	0.04 (JH)	E	[34]
Archer	3D	0.40 (l)	3.10	0.63 (JH)	E	[35]
5 cm Monpond	Micro	0.05×0.05×0.05	0.030	0.001 (JH)	E	[38]
Yang’s robot ^a^	Micro	0.14 (H)	0.048	0.58 (JH)	E	[39]
PogoDrone	Micro	0.15 (H), 0.09 (W)	0.031	0.70 (JH)	E	[42]
Hopcopter	Micro	0.22 (H), 0.10 (W)	0.035	1.63 (JH)	E	[43]

C, classification; D, dimension (m); M, mass (kg); l, leg length (m); L, length (m); W, width (m); H, height (m); JH, jumping height; Fv, forward speed (m/s); P, pneumatic; E, electric; Hy, hydraulic; HF, hydrocarbon fuel

^a^ First author’s name was attributed to robot.

The telescopic configuration is most commonly applied in bipedal robots. For instance, a parallel robot with 2 telescopic legs and a connected torso was proposed by Moscow University [Fig. 2(4c)] [48]. Each telescopic leg consists of a thigh and a calf, with the calf sliding along an internal iron rail within the thigh, thereby changing the total leg length and adjusting the gait and mobility. In 2001, Kajita et al. [49] proposed a new bipedal vehicle with telescopic legs named Meltran V [Fig. 2(4d)]. It moves by adjusting the distance between the hip and ankle joints by telescoping the polygonal joints, driven by a DC servo motor and a ball screw. Furthermore, Mou et al. [50] proposed the humanoid robot L04 [Fig. 2(4h)], where each leg is driven by 2 Maxon RE40 motors. These motors independently control the vertical telescoping of 2 sliders, simulating knee bending and adjusting the leg height through leg extension.

Compared to bipedal robots, quadruped, hexapod, and other MLRs exhibit more complex movement patterns. However, the limited DoF inherent in telescopic leg configurations constrain their ability to execute intricate tasks. Consequently, only a limited number of prototypes have adopted this design. A notable example is the classic quadrupedal robot “Scout II”, which adopts a telescopic configuration with a compliant prismatic structure with steel springs [Fig. 2(4e)] [51,52]. Each leg unit is composed of 2 parts connected by a spring, forming a compliant prismatic joint that allows the robot to achieve a certain degree of compliance during motion. Rushworth et al. [53] introduced a walking parallel kinematic machine called WalkingHex [Fig. 2(4f)], which is controlled by 3 cables connected at intervals. The WalkingHex can move at a speed of 0.2 m/min, walk up to 15 m, and execute a gait with a stride length of 40 mm. Furthermore, Mohamed et al. [54] presented a hexapod robot featuring telescopic legs connected to pivot joints at the hip [Fig. 2(4g)]. Each leg is equipped with a thin rubber layer to improve frictional contact.

#### Summary of the telescopic SLRs

Telescopic SLRs feature a relatively small workspace and a straightforward control strategy during motion, effectively reducing collision risks with the environment obstacles. These advantages were especially beneficial in early research on dynamic hopping. However, despite their comparatively favorable performance in basic mobility tasks, the telescopic configuration presents challenges such as limited DoF and restricted foot placement capability. Consequently, telescopic SLRs face challenges in adapting to terrain changes or diverse movement patterns during more complex jumping tasks. Overcoming these limitations will require either design improvements or the integration of additional technologies.

### Articulated SLRs

**Fig. 3.**
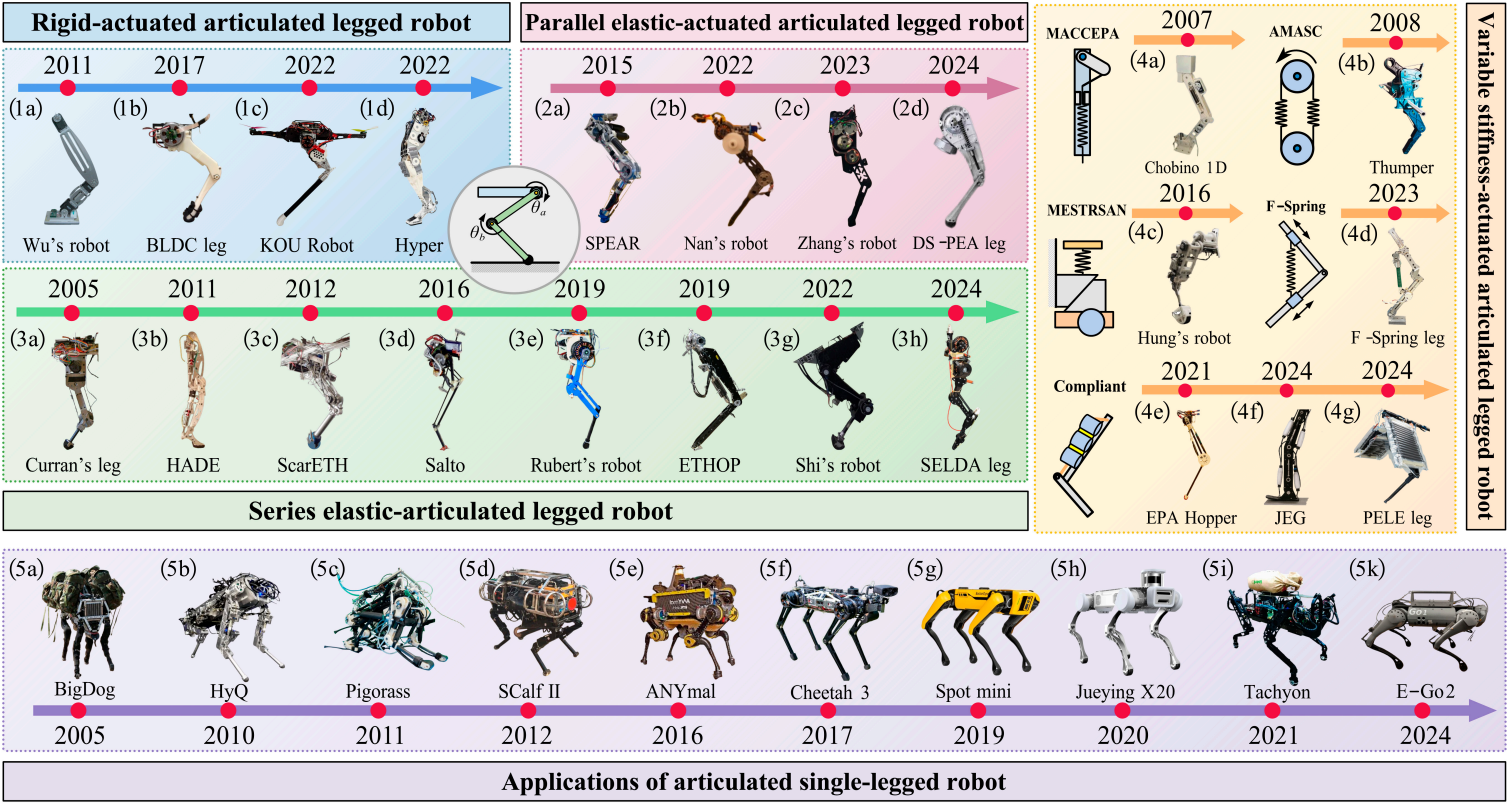
Categorization and application domains of articulated SLRs.

Inspired by the dynamic movement patterns of highly dynamic animals (e.g., cats, dogs, and cheetahs), researchers have proposed articulated SLRs typically consist of multiple linkages connected by active or passive rotational joints, such as the hip, knee, and ankle joints. The incorporation of multiple DoFs in the joints allows these robots to execute more complex and flexible motion trajectories [4]. Since the 1990s, research on articulated SLRs has gained increasing attention in the robotic field. The classification and applications of articulated SLRs are shown in Fig. 3.

#### Classification of articulated SLRs

**Fig. 4.**
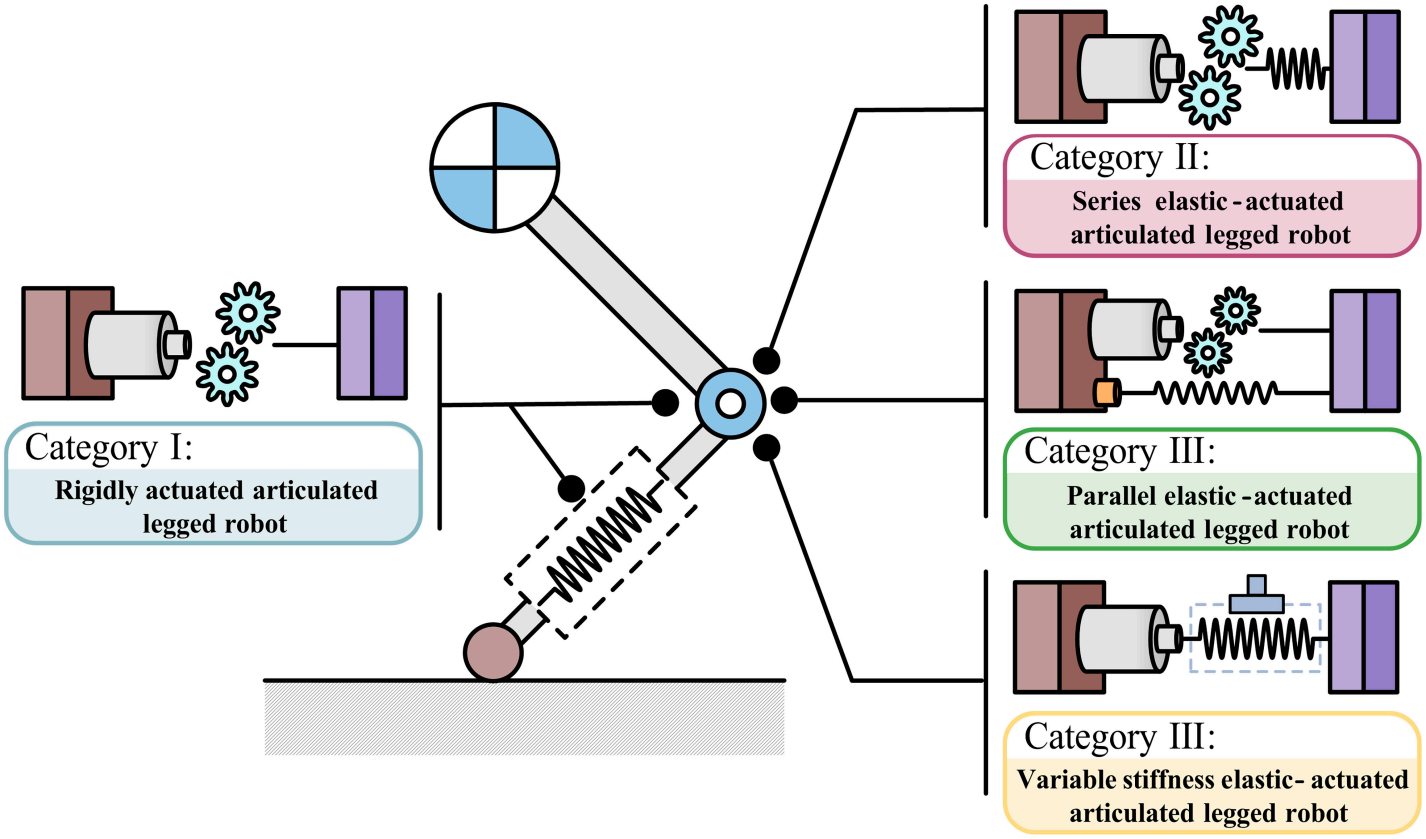
Characteristics of 4 types of articulated SLR.

According to the different types of internal actuator, articulated SLRs can be classified into rigid-actuated articulated legged robot (RALR), parallel elastic-actuated articulated legged robot (PEALR), series elastic-actuated articulated legged robot (SEALR), and variable stiffness elastic-actuated articulated legged robot (VSELR), as shown in Fig. 4.

##### Rigid-actuated articulated legged robots

RALRs are driven by rigid actuators without elastic components like springs or torsion springs. This configuration features a relatively simple mechanical structure, facilitating manufacturing and maintenance. Furthermore, the absence of elastic elements in the actuators simplifies the establishment of the dynamic model, thereby enabling high-precision motion control.

Wu et al. [55] proposed a robot driven by 2 servomotors at the ankle and the hip joints [Fig. 3(1a)]. A distinctive feature of this design is that the foot rotates around the nondriven toes before takeoff, enabling the robot to perform stable, human-like hops over various distances on flat ground. Furthermore, the robot can successfully navigate stairs, demonstrating enhanced locomotion versatility. In 2017, a robot was built by Ding and Park [56], which is driven by 2 coaxially arranged brushless motor modules combined with a planetary gearbox [Fig. 3(1b)]. The robot utilizes the advantages of high-torque-density electromagnetic actuators and low gear ratio transmissions, achieving a maximum vertical jumping height of 0.62 m and a maximum forward jumping distance of 0.72 m. Zeng et al. [57] developed a planar robot utilizing rotor-assisted locomotion named KOU [Fig. 3(1c)]. The design integrates the entire thigh into the output section of the cycloidal gear reducer, improving stiffness and compactness. Inspired by human biomechanics, Kim et al. [58] proposed a robot called HyperLeg [Fig. 3(1d)], featuring a 1-DoF knee joint, a 2-DoF ankle joint, and a 1-DoF toe joint. All actuators are located at the proximal part of the thigh frame to minimize the distal mass, aiming to achieve high backdrivability and joint stiffness. Bakırcıoğlu et al. [59] applied evolutionary algorithms to optimize structural parameters, leading to the design and fabrication of a hydraulically actuated legged robot with rigid transmission. The robot demonstrates high-precision trajectory tracking capability and achieves a force transmission efficiency as high as 94%.

Purely rigid-driven robots require a continuous power supply from the actuators to maintain stable standing postures. Additionally, rigid collisions with ground often cause substantial energy loss, greatly limiting the energy efficiency and endurance of RALRs [60]. Inspired by the muscle and tendon structure of mammals, researchers have begun to explore the incorporation of passive elastic joints into rigid actuators to store energy generated during landing and reuse it for subsequent movements. Early examples, such as the “Monopod”, incorporated a spring between the foot and calf to maintain tendon tension [61]. Similarly, the hydraulic-driven robot KenKen, designed by Hyon and Mita [62], based on the bionic structure of canine hind legs, features a spring-passive ankle joint. The passive joints reduce the impact when the foot contacts the ground and enable the release of kinetic energy during the landing process. However, the introduction of passive elastic joints increases the complexity of the leg structure and limits the ability to achieve precise control [63].

##### Parallel elastic-actuated articulated legged robots

Researchers have integrated elastic components into actuators to effectively store and release energy. This integration simplifies the overall structure while ensuring improved dynamic stability and motion efficiency of the robot [64,65]. One approach involves parallelizing the elastic elements with the actuator without altering the integrity of the original mechanism [66].

The PEALRs utilize the parallel elastic actuator (PEA) for actuation, where the elastic elements efficiently provide the necessary torque required. This significantly reduces the peak actuator torque and energy consumption, which enables the robot to utilize more compact driven units [67,68]. Additionally, PEALRs could directly regulate the output torque of the end actuator, markedly improving the control bandwidth and enhancing the dynamic response capability [69,70].

Zhang et al. [71] leveraged the gas spring combined with a cam roller to generate a customizable torque compensation profile for the robot [Fig. 3(2c)]. By adjusting the knee joint angle, the cam module modifies the distance to the center of rotation, thereby modulating the compression of the gas spring to compensate for output torque. The implementation of the mechanism reduces energy consumption. In a related approach, Liu et al. [72] designed a compact dual-slide robot incorporating a compression spring structure [Fig. 3(2d)]. By employing the dual-slider mechanism, the circular motion of the knee joint is decomposed into vertical motion along the linkage, which compresses the spring to store energy. This design not only reduces overall energy consumption but also enhances structural compactness and mechanical efficiency.

However, PEAs typically introduce nonlinear spring force and torque characteristics. When the compliant elements are engaged with the actuator, the actuator must counteract the tensile or compressive forces of the spring to generate output [73]. To overcome this drawback, the concept of inserting a switchable spring in parallel with an actuator has been mentioned in Ref. [74], allowing the elements to engage or disengage based on motion requirements. Liu et al. [75,76] proposed a robot driven by a switchable PEA named SPEAR [Fig. 3(2a)]. The mechanical switch engages the spring during support phase to provide the necessary support force. Conversely, during the flight phase, the spring disengages to allow free joint movement. The switchable design enhances energy efficiency without compromising the locomotion performance. Inspired by the biomechanics of flamingos, Nan et al. [77] proposed a reconfigurable legged robot [Fig. 3(2b)] that utilizes regions within the joint space to enhance joint adaptability. This design achieves switching by crossing the singularity of the leg, eliminating the need for additional actuators to control elastic engagement.

While the interference of elastic elements can be partially mitigated by incorporating additional switchable actuators, the added actuators introduce extra weight and complexity to the structure, thereby limiting overall mobility improvements. More importantly, the inclusion of mechanical switches increases the complexity of the control system, necessitating more precise adjustments and real-time feedback mechanisms.

##### Series elastic-actuated articulated legged robots

In addition to PEA, another widely utilized actuator configuration integrates a compliant element in series with the driven system, forming a SEA [78,79]. SEAs are renowned for their superior force control precision compared to PEA during actuation [80]. Additionally, series elastic elements can more effectively absorb substantial impact forces by mitigating shock loads during ground contact [81].

Curran [82] proposed a lightweight SEALR [Fig. 3(3a)] powered by a motor and a cable–pulley transmission system. In the knee joint actuation mechanism, the motor adjusts the rest position of a torsion spring, thereby transmitting torque to the lower leg linkage via the spring. Hutter et al. [83] from ETH Zurich designed a robotic leg named ScarETH [Fig. 3(3c)], aimed at enhancing the jumping capability of running robots. ScarETH incorporates 2 SEAs to independently control the flexion and extension of the hip and knee joints, thereby reducing overall energy consumption.

To further enhance the locomotion capabilities of SEALRs, researchers have drawn inspiration from the structure and movement patterns of animals. For instance, the hybrid actuator development (HADE) robot [Fig. 3(3b)] is inspired by horse leg design [84]. It features hip, knee, and ankle joints powered by “SEA 23-23”, with an additional magnetic damping integrated into the knee joint to provide variable damping throughout the gait cycle. HADE achieves a compliant, natural motion in an energy efficient manner. Inspired by the galago, the Salto robot [Fig. 3(3d)] further enhances jumping agility by integrating series elastic elements with a mechanical advantage profile designed limb [85]. The motor with a conical torsional latex spring [86] adjusts the limb to generate thrust against the ground. The prototype achieved a maximum jump height of 1.008 m, held the record for the highest vertical jumping agility prior to 2016. The SEALR imitating the human muscles [Fig. 3(3e)] was proposed by Ruppert and Badri-Spröwitz [87], utilizing 2 elastic elements to provide compliance for movement. The knee spring simulates the quadriceps and patellar tendon in natural organisms, while the lower leg spring emulates the gastrocnemius and Achilles tendon. This configuration reduced the average torque required for the robot with a double joint spring by 74%.

In addition to the conventional applications discussed above, Bolignari et al. [88] also applied the SEA to the robot’s ankle joint [Fig. 3(3h)], driven by a lightweight series elastic diaphragm distal actuator (SELDA) that can be approximated as a linear spring. The SELDA ankle joint enhances the motion performance of the robot, increasing its forward velocity by 93%. While electric robots commonly adopt SEA, hydraulic robots rarely applied SEA due to the high torque generated at the joints dealing with heavy loads. Harbin Institute of Technology [89] proposed a hydraulic legged robot that utilizes the SEA principle shown in Fig. 3(3g), considering mechanical structure, weight, and dimensional constraints. The addition of elastic elements effectively enhances compliance and mitigates ground impact force.

However, conventional SEALRs typically utilize elastic elements only during the support phase, where the spring counteracts the ground reaction forces. The series elasticity cannot be effectively utilized in the absence of ground reaction forces. To address this limitation, researchers have attempted to incorporate discrete coupling mechanisms in SEAs, allowing the spring to be preloaded during the flight phase [90]. One such approach was proposed by Guenther et al. [91], who introduced a robot named ETHOP [Fig. 3(3f)], which employs a ratchet mechanism with an actuated pawl to disconnect and link the knee joint elastic elements, ensuring high performance and precise control of preloaded frequency hopping.

PEALRs exhibit superior energy efficiency during periodic movements such as squatting and walking, while also reducing peak torque requirements. Consequently, for applications involving repetitive motion patterns and stringent energy efficiency demands, PEALRs represent an ideal actuation solution. By contrast, SEALRs offer better performance in short-duration, high-intensity activities such as explosive jumping. In addition to the enhanced capacity for impact absorption, SEALRs enable more precise force control. Therefore, they are particularly well-suited for scenarios requiring high force control accuracy or operating under conditions involving substantial external impacts. Therefore, the selection of an appropriate actuator configuration should be based on the specific task demands and operational environments.

However, robotic legs require varying stiffness to maintain stability during running and jumping, similar to the way animals modulate their limb stiffness based on the task demands [92,93]. The stiffness of the traditional SEA is typically fixed in the design process of elastic components, which limits the maximum operating speed and the effective load [94,95]. Robots with fixed stiffness face challenges in performing various tasks; therefore, variable stiffness designs are increasingly desirable in robotic legged locomotion systems.

##### Variable stiffness elastic-actuated articulated legged robots

Researchers have introduced various actuators capable of controlling both their balance positions and stiffness [96,97] to enhance the performance of legged robots, paving the way for the next generation of VSELRs.

The mechanically adjustable compliance and controllable equilibrium position actuator (MACCEPA) is a type of electric actuator that drives the lever arm to rotate and stretch the cable between the fixed point, adjusting the length of the rope between the spring and the fixed point [98]. Vanderborght et al. [99] enhanced the conventional MACCEPA design by replacing the lever arm with a profile disk, enabling refined adjustment of the torque angle. This modified configuration was tested on the robot named Chobino1D [Fig. 3(4a)]. The resulting actuator exhibited stiffness that increased with angular deflection, replicating the nonlinear elastic behavior of biological tissues.

The actuator with mechanically adjustable series compliance (AMASC) stretches 2 identical opposing springs through an additional motor, allowing for the modulation of the stiffness in the system’s natural dynamics [100]. Hurst and Rizzi [101] designed and constructed a prototype named Thumper [Fig. 3(4c)] based on the construction and simulation development ideas of AMASC. Thumper employs 2 antagonistic springs and a small brake motor for joint stiffness adjustment, achieving a maximum height of 1.05 m and performing periodic jumps.

The L-MESTRAN actuator modulates joint stiffness by driving a rack gear that compresses an internal spring through interaction with an inclined gear surface [102]. This design allows dynamic tuning of the knee joint stiffness in accordance with varying stride frequencies, thereby optimizing energy efficiency across different hopping conditions. Moreover, it utilizes a non-backdrivable worm-gear transmission, enabling the maintenance of the desired stiffness level without continuous energy input.

The floating spring actuator (F-Spring) modulates stiffness by moving the endpoint of a floating spring using a self-locking slider, which can be fixed at designated positions [103]. This mechanism enables energy storage under a low-stiffness configuration and then transitions to a high-stiffness state via a lockable spring, thereby amplifying the output force. Experiments demonstrate that the F-Spring-equipped robotic leg can successfully perform a squat-to-stand task under a 27-kg load, which cannot be achieved using fixed stiffness springs [104].

In addition to the methods previously mentioned, several newly developed types of variable stiffness actuators (VSAs) have also been applied to VSELRs, such as the following: the VLLSA in Ref. [105] employs a roller-bearing slider to modify the effective length of a steel plate spring; the VS actuator in Ref. [106] utilizes 3 pairs of gear-rack mechanisms to achieve stiffness modulation; and the shape memory alloy (SMA)-based actuator in Ref. [107] leverages shape memory alloys to preadjust its length in response to external loads, thereby altering the stiffness.

Although most VSAs are capable of adapting to external environments, their motion efficiency still falls short of that observed in biological muscles. Artificial muscles, which represent the closest analogues to their biological counterparts, are actuated by external stimuli such as fluid pressure or electromagnetic signals to produce push–pull motions [108,109]. Sharbafi et al. [64] proposed the EPA Hopper [Fig. 3(4e)] that utilized pneumatic artificial muscles (PAMs) as parallel extensor and flexor muscles [Fig. 3(4e)]. Jumping experiments showed that both PAMs reduced peak torque and power during stance and flight phases. Subsequently, recognizing that the ankle joint more closely resembles human elastic movement behavior, a curved foot and additional PAMs for ankle extension were incorporated to enhance the EPA Hopper [110]. The integration of PAMs in the enhanced EPA Hopper facilitates high-performance jumping with reduced energy consumption, achieving a maximum jump height of 30 cm. This configuration greatly improves the robot’s ability to regulate energy dynamics throughout the locomotion cycle.

The combination of actuators and elastic pneumatic muscles offers the flexibility to adapt to complex environments, but electromagnetic motor drive systems remain inherently complex to control [111]. Another type of VSELR directly driven by artificial muscles can pave the way for more flexible, adaptive, and energy-efficient robots to move and operate in natural environments.

Liow et al. [112] proposed a novel robotic leg design in which the structural body is composed of lightweight aluminum frames, reinforced with 3D-printed acrylonitrile styrene acrylate brackets to substantially reduce overall mass. The joints are actuated by a combination of belt transmissions and fiber-jamming tendons. This compliant actuation approach not only mitigates impact forces during joint motion but also exploits the intrinsic stiffness and damping characteristics of the tendons to enhance joint-level compliance. Buchner et al. [113] proposed a flexible robot named “PELE”, which is driven by hydraulic amplification self-healing electrostatic actuators [Fig. 3(4f)]. The PELE robot surpasses traditional actuator systems by achieving high energy efficiency (1.2% of typical consumption), agile locomotion with >5 Hz gait cycles and 128 mm jumps, stable movement on unstructured terrains via open-loop control, and intrinsic capacitive sensing for real-time joint state and disturbance detection without encoders. 

Despite these innovations, the addition of mechanisms can also make the overall structure complex and cumbersome. Furthermore, the nonlinear characteristics and heightened system complexity of VSAs pose considerable challenges for precise joint control of SLRs.

Table 3 indicates the key important articulated SLRs, their physical parameters, and mobility.

**Table 3.** Structural parameters of typical articulated SLRs

Name	C	Mobility	DoF	*D*/m	*M*/kg	Actuator	Refs.
Wu’s robot ^a^	RALR	0.20 (FD)	3	0.28 (T), 0.17 (S), 0.08 (F)	0.87	DC servomotors with gearbox	[55]
BLDC leg	RALR	0.62 (JH), 0.72 (FD)	3	0.12 (T), 0.12 (S)	0.64	Electromagnetic actuators with gearbox	[56]
KOU	RALR	0.21 (JH), 0.36 (FD)	2	0.12 (T), 0.23 (S)	1.20	Motors with cycloidal gearbox	[57]
HyperLeg	RALR	0.79 (JH), 0.90 (FD)	4	0.35 (T), 0.36 (S), 0.18 (F), 0.08 (To)	8.16	Motors with planetary gear and timing pulley	[58]
Bakırcıoğlu’s robot ^a^	RALR	/	2	0.40 (T), 0.40 (S)	6.12	Linear hydraulic cylinders	[59]
KenKen	RALR	0.60 (JH), 2.00 (FD)	3	0.18 (T), 0.18 (S), 0.05 (F)	3.60	Linear hydraulic cylinders	[62]
SPEAR	PEALR	0.64 (JH), 0.54 (Fv)	2	0.32 (T), 0.33 (S)	8.07	Series elastic actuator with cable–pulley system	[75,76]
Nan’s robot ^a^	PEALR	0.42 (JH)	2	0.37 (L)	0.83	Motor with 2-stage transmission system and clutch mechanism with the spring	[77]
Zhang’s robot ^a^	PEALR	/	2	0.25 (T), 0.25 (S)	3.90	Motor with cycloid reducer, belt system, gas-spring and cam module mechanism	[71]
DS-PEA leg	PEALR	0.50 (JH)	3	0.22 (T), 0.2 (S)	2.24	Motor with gearbox and dual-slide PEA	[72]
Curran’s robot ^a^	SEALR	0.75 (JH)	2	0.14 (T), 0.14 (S)	4.02	Motor with gearbox and torsion spring	[82]
HADE	SEALR	0.54 (Fv)	3	0.51 (T), 0.46 (S), 0.21 (F)	11.00	Series elastic actuator combined with MRF rotary damper	[84]
ScarlETH	SEALR	0.25 (FD), 0.37 (JH)	2	0.20 (T), 0.20 (S)	6.20	Motor with harmonic reducer, micro chain, pulley system, and compression spring	[83]
Salto	SEALR	1.01 (JH)	2	0.15 (L)	0.10	Motor with torsional spring and linkage mechanism	[85]
Rubert’s robot ^a^	SEALR	0.49 (JH)	3	0.15 (T), 0.15 (S), 0.15 (F)	0.91	Motors with gearbox and spring	[87]
ETHOP	SEALR	0.40 (JH)	2	0.60 (T), 0.64 (S)	14.70	SEA combined with ball screw and ratchet mechanism	[91]
Shi’s robot ^a^	SEALR	/	2	0.33 (T), 0.33 (S)	/	Hydraulic cylinder and elastic element	[89]
SELDA leg	SEALR	0.73 (FD), 0.24 (JH)	3	0.15 (T), 0.15 (S), 0.14 (A), 0.07 (F)	1.20	Motor with gearbox and SELDA system	[88]
Chobino1D	VSELR	0.07 (Jv), 1.20 (Fv)	1	0.2 (T), 0.2 (S)	/	MECCPA2.0 actuator with cable system	[99]
Thumper	VSELR	1.05 (JH)	2	0.5 (T), 0.5 (S)	30.00	Motor with spring and differential	[101]
Hung’s robot	VSELR	1.25 (Fv)	2	0.12 (T), 0.16 (S)	1.00	Motor with gearbox and worm-gear	[102]
F-Spring leg	VSELR	/	5	/	8.20	Motor with floating spring mechanism	[104]
EPA Hopper	VSELR	0.10 (JH)	2	0.27 (T), 0.27 (S)	3.00	Electric motor and pneumatic muscle	[64]
JEG	VSELR	/	3	/	3.65	Servo motor with belt transmission system and antagonistic tendons	[112]
PELE Leg	VSELR	0.13 (JH), 0.75 (Jv)	2	0.28 (T), 0.28 (S)	0.23	Peano-HASEL actuators	[113]

C, classification; D, dimension (m); M, mass (kg); FD, forward distance (m); JH, jumping height (m); Fv, forward velocity (m/s); Jv, jumping velocity (m/s); T, thigh length (m); S, shank length (m); F, foot length (m); A, ankle length (m); To, toe length (m); L, extended leg length (m)

^a^ First author’s name was attributed to robot.

#### Applications of articulated SLRs

The articulated leg configuration has become the predominant structural design in MLRs, primarily due to its significant technological advantages in motion performance afforded by multiple freedoms. This configuration enables exceptional flexibility and high-speed locomotion, allowing robots to effectively traverse both individual terrain types and complex, mixed environments.

BigDog [Fig. 3 (5a)] was developed by Boston Dynamics [114] for military applications over complex and rough terrains. It has excellent wilderness adaptability, capable of walking on snow, gravel, and muddy paths with a weight of 153 kg. Subsequent research in universities and research institutes expanded the study of articulated MLRs. The Italian Institute of Technology [115] built a hydraulically and electrically actuated robot “HyQ” [Fig. 3(5b)], aimed at highly dynamic motions across different types of terrains. The robot has a height of 0.98 m and a weight of approximately 80 kg in standing mode [116]. Yamada et al. [117] designed a simple quadruped robot “Pigorass” [Fig. 3(5c)] that employed PAMs, which are powered by an external air compressor and controlled via proportional pressure-control valves to regulate internal pressure. Later in 2012, the team at Shandong University introduced Scalf-II [Fig. 3(5d)] [118], with dimensions of 1.1 m × 0.45 m × 1.1 m. The leg structure incorporates 3 rotational joints, enabling hip adduction/abduction, hip flexion/extension, and knee flexion/extension driven by hydraulic cylinders [119]. The Anymal robot proposed in 2016 [Fig. 3(5e)] has dimensions of 0.8 m × 0.4 m × 0.7 m and weighs 30 kg [120]. It is primarily designed for industrial applications and operation in hazardous environments. Each leg is composed of 3 joints, which can adapt to complex tasks through folding and changing configurations. Cheetah 3 [Fig. 3(5f)], developed by the MIT Biorobotics Lab [121,122], is the third-generation robot in the Cheetah series. The length of each leg is 0.34 m, which includes a self-made high-torque density proprioceptive actuator and a reversible planetary gear reducer, enabling agile swinging and twisting motions with high precision and responsiveness.

Following the successful development of MLRs in academic institutions, subsequent generations of MLRs after BigDog developed by Boston Dynamics [123] have shifted focus from military applications to more industrial and service-oriented robotics applications. Spot [Fig. 3(5g)] has dimensions of 1.1 m × 0.5 m × 0.84 m and a maximum speed of 5.76 km/h. With a total weight of 30 kg, it can carry a payload of 14 kg. All joints are electrically driven and powered by batteries, offering omnidirectional walking and running capabilities [124]. Jueying X20 [Fig. 3(5h)] is a mid-sized (0.95 m × 0.47 m × 0.7 m) and mid-weight (50 kg) robot with a maximum speed of 4.95 m/s and a payload capacity of up to 85 kg [125]. It can operate for 2 h under a 20-kg load and for 4 h without any load. Researchers from Sony Group Corporation proposed a robot “Tachyon” [Fig. 3(5i)] [126]. Featuring a novel compact series-PEA (SPEA) on the upper link and a 4-bar linkage design in the knee joint, Tachyon can carry payloads exceeding 20 kg while maintaining dynamic walking performance. Ding et al. [127] introduced an easily accessible quadruped “Delft E-Go” [Fig. 3(5k)] based on the product Unitree Go1, which is capable of walking forward at a maximum speed of 0.25 m/s, and walking diagonally at 0.1 m/s. The detailed completion reports for the MLRs are summarized in Table 4 as per the availability of data.

**Table 4.** Specifications of traditional MLRs

Name	Year	*D*/m	*M*/kg	PL/kg	*A*	*V*/m·s	Scene	C	DoF	Country
BigDog	2005	1.00×0.30×1.00 (*L*×*W*×*H*)	109	154	Hy	3.50	Wartime material transportation	RAL	4	America
HyQ	2010	1.00×0.50×0.98 (*L*×*W*×*H*)	90	/	Hy/E	2.00	Highly dynamic tasks in complex terrain	SEAL	3	Italy
Pigorass	2011	350.00 (*L*)	4	/	PAM	0.66	Scientific research	VSEL	3/2	Japan
SCalf II	2012	1.10×0.45×1.10 (*L*×*W*×*H*)	120	75	Hy	1.28	Scientific research	RAL	4	China
ANYmal	2016	0.80×0.60×0.70 (*L*×*W*×*H*)	32	10	E	1.00	Highly dynamic tasks in complex terrain	SEAL/PEAL	3	Germany
Cheetah 3	2017	0.60×0.26×0.20 (*L*×*W*×*H*)	45	/	E	13.30	Scientific research	RAL	3	America
Spot mini	2019	1.10×0.50×0.84 (*L*×*W*×*H*)	30	14	E	1.60	Market produce	RAL	3	America
Jueying X20	2020	0.85×0.50×0.65 (*L*×*W*×*H*)	40	10	E	1.94	Market produce	RAL	3	China
Tachyon	2021	/	41	20	E	0.15	Scientific research	SPEAL	3	Japan
E-Go	2024	0.59×0.22×0.29 (*L*×*W*×*H*)	13	/	E	0.25	Scientific research	PEAL	3	Netherlands

*D*, dimension (m); M, mass (kg); PL, payload (kg); A, actuator; V, speed (m/s); C, classification; L, length (m); W, width (m); H, height (m); Hy, hydraulic; E, electric; PAM, pneumatic artificial muscle

#### Summary of the articulated SLRs

Articulated SLRs adopt a biomimetic joint structure, incorporating hip, knee, ankle, and other joints to replicate the movement of biological limbs, enabling the robot to execute more intricate motion trajectories through coordinated multi-DoF joint movements. The advantages and disadvantages of different types of SLRs are summarized in Table 5. Articulated SLRs are capable of executing complex motion patterns and adapting to different terrains, such as rocky surfaces, stairs, and other intricate environments. However, the high DoFs of the leg structure result in increased mechanical design and manufacturing costs. Moreover, effective control demands precise coordination and synchronization among multiple actuators, thereby increasing the complexity.

**Table 5.** Advantages and limitations of articulated SLR variants

Type	Advantages	Limitations
RALR	1. Simple structure and implementation 2. Easy to achieve precise control	1. Impact contact forces affect the internal structure 2. Inelastic collisions result in high energy-consumption demands
PEALR	1. Greatly reduces the peak torque requirements 2. Enhanced energy efficiency 3. Achieves higher control bandwidth, effectively reducing the lag and overshoot phenomena	1. Unstable torque causes nonlinear behavior, compromising precise control
SEALR	1. Buffer impact force when landing 2. Substantial improvement in energy efficiency during highly dynamic locomotion	1. Complex structure 2. Fixed stiffness limits the maximum operating speed and the effective load
VSELR	1. Easily adapts to different terrains and task requirements 2. Improved motion efficiency and adaptability	1. Complex and cumbersome overall structure 2. Difficult to achieve precise motion control

### Challenges in SLR structural design

To facilitate a systematic understanding of structural diversity, the comparative analysis of representative SLR configurations is shown in Table 6.

**Table 6.** Comparative analysis of different structural configurations in SLRs

Type	Subtype	Features	Typical application scenarios
Telescopic SLR	2D telescopic SLR	Simple planar structure with linear extension; vertical or sagittal plane hopping.	Basic locomotion experiments; theoretical model validation.
	3D telescopic SLR	Capable of spatial hopping and pose control via universal joint; provide terrain adaptability.	Rough-terrain exploration; 3D theoretical model validation.
	Micro telescopic SLR	Miniaturized form factor (<200 g); compact scale with short actuation stroke; suitable for ultra-high horizontal jumping.	Disaster response; military reconnaissance; insect-scale bioinspired platforms.
Articulated SLR	RALA	Rigidly actuated with articulated joints; partially incorporating elastic passive joints; high control accuracy; structurally vulnerable to impact damage.	Early-stage limb design; precise trajectory tracking; rigid-body dynamics research.
	PEALR	Parallel elastic actuator integration; notably reduces energy consumption and peak torque; affected by elastic reaction forces.	Repetitive motion tasks; applications with strict energy efficiency requirements.
	SEALR	Series elastic actuators integration; impact buffering capability; high agility and explosive power.	High-intensity activities, lightweight structural systems, common structural configuration for MLRs.
	VSELR	Variable stiffness actuators integration; stiffness modulation for energy tuning and compliance control.	Complex and unstructured terrains; adaptive control platforms; future research in legged robotics.

In the structural design of SLRs, achieving an optimal balance among mechanical strength, dynamic stability, and actuation performance, while simultaneously minimizing weight and geometric dimensions, remains a fundamental challenge. Compact structural layouts often conflict with the spatial requirements of embedded sensors, actuators, and transmission systems, thereby constraining the system’s functional integration. Moreover, during dynamic operations such as jumping and running, SLRs are subject to repeated high-impact loads, which impose substantial mechanical stress on the leg structures. As a result, ensuring long-term structural reliability requires careful attention to material fatigue behavior, joint reliability, and stress distribution, especially under prolonged operational cycles in harsh environments. Environmental adaptability further complicates structural design. In real-world or unstructured settings, robots must cope with dust, moisture, temperature fluctuations, and irregular terrain, often without relying on extensive sealing or external protection [128,129].

In addition, the transition from SLRs to MLRs introduces a new level of structural and system-level complexity. As the number of limbs increases, the total DoFs grow multiplicatively, raising the risk of spatial interference between limbs during dynamic motion. This demands more sophisticated joint layout optimization, collision avoidance strategies, and symmetric structural configurations to ensure reliable operation. Simultaneously, the perception system must integrate multimodal sensory inputs across all limbs to achieve accurate estimation of whole-body states, including body posture, leg kinematics, and ground interaction status. These requirements place substantial demands on system architecture, particularly in terms of real-time computation, subsystem coordination, and data fusion capability, thereby elevating the complexity and performance thresholds of both hardware and control algorithms.

## Control Strategy for SLRs

### Modeling and simulation of SLRs

**Fig. 5.**
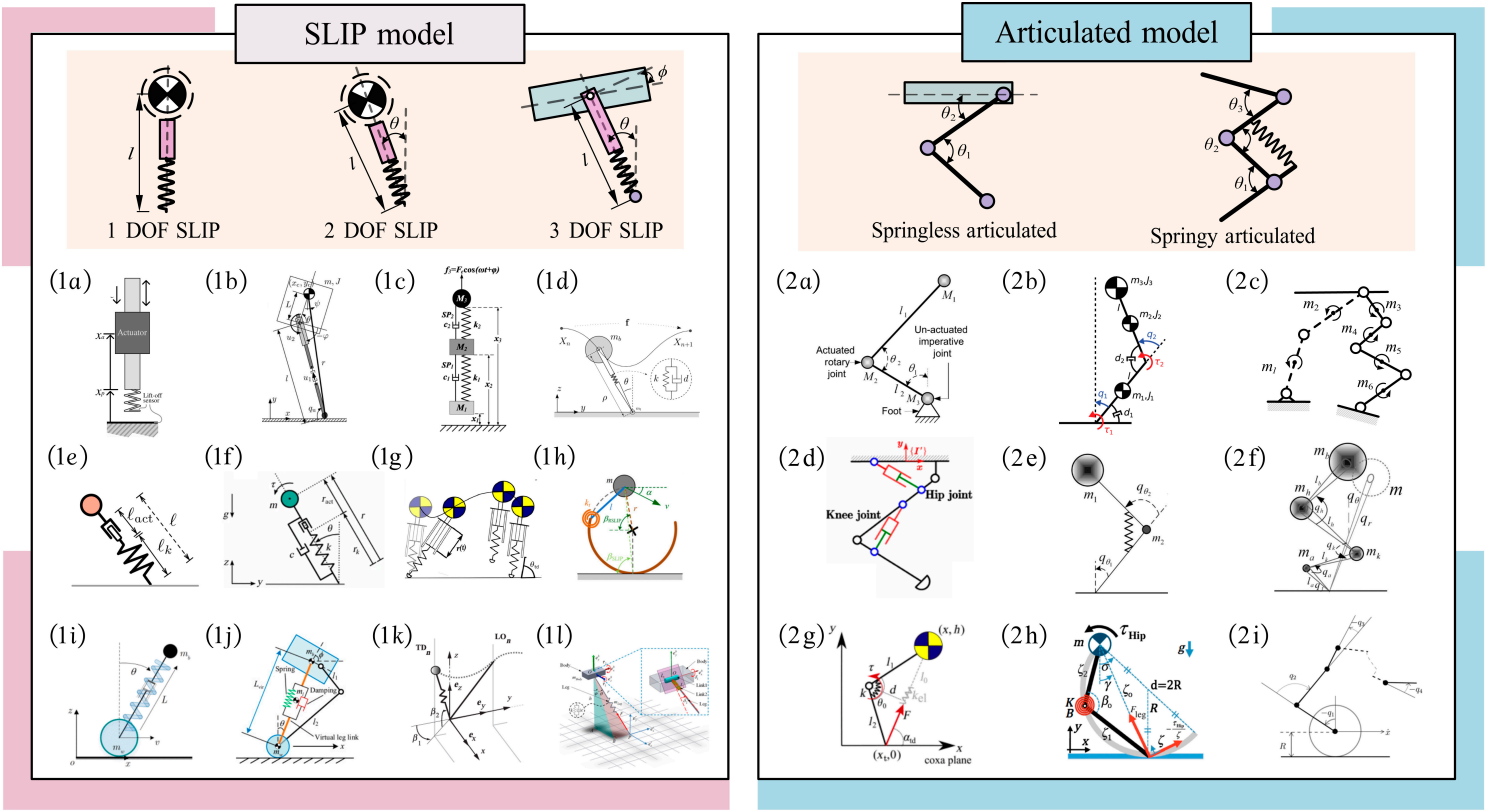
Overview of modeling approaches for SLRs: (1a) 1-DoF SLIP model, (1b) A-SLIP model, (1c) three-mass SLIP model, (1d) D-SLIP model, (1e) active-SLIP model, (1f) MD-SLIP model, (1g) U-SLIP model, (1h) R-SLIP model, (1i) W-SLIP model, (1j) W-LS-LIP model, (1k) Seipel’s SLIP model, and (1l) 3D-SLIP model; (2a) Berkemeier’s model, (2b) Roozing’s model, (2c) VDC model, (2d) closed-chain VDC model, (2e) SLSK model, (2f) AKF model, (2g) U-articulated model, (2h) He’s model, and (2i) Chen’s model.

Compared to traditional wheeled or tracked robots, SLRs feature a more complex multi-DoF structure, resulting in the modeling process being more intricate and challenging. Establishing accurate models is essential, which serves as the foundation for developing control strategies. Commonly, 2 types of modeling approaches have been commonly adopted in SLRs, namely, the SLIP model and the articulated model based on structural simplification, as shown in Fig. 5.

#### SLIP model

**Fig. 6.**
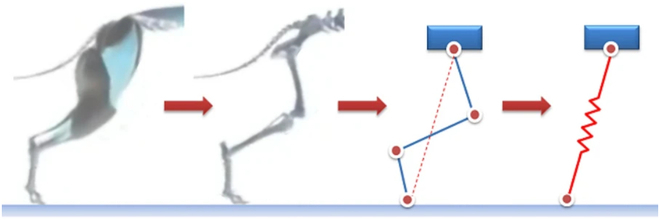
Equivalent modeling process of SLRs [151].

SLIP models have been extensively utilized in the study of locomotion in quadrupedal organisms due to their conceptual simplicity and effectiveness in capturing the fundamental dynamics of individual leg behavior [130,131]. Initially proposed by Blickhan in 1989 [132], the SLIP model analogizes the muscle and tendon structure of mammalian legs to a massless spring system, with the remaining mass of a single leg unit concentrated at the load point at the top of the spring, as shown in Fig. 6.

Considering different motion ranges during modeling, SLIP models can be categorized into three: the 1-DoF SLIP model, the 2-DoF SLIP model, the 3-DoF SLIP model.

The 1-DoF SLIP model only accounts for the extension and contraction DoFs along the spring direction, reflecting the in-place hopping motion of SLRs in a simple and intuitive way. For example, Aguilar et al. [133] proposed a 1D mass-spring model with an active driving mass [Fig. 5 (1a)], which compresses the spring through the driver to study the self-deformation of the leg during motion.

The 2-DoF SLIP model introduces an additional swinging freedom between the spring and the mass block to achieve planar motion. The model is widely adopted in research and practical applications due to its ability to better represent more complex movements, especially in dynamic running and hopping behaviors.

In the traditional 2-DoF SLIP model, the center of mass (COM) is assumed to lie along the extension line of the spring, which cannot accurately represent the scenario of robots with asymmetric mass distributions. To address this, Poulakakis and Grizzle [134] proposed an asymmetric SLIP model [Fig. 5 (1b)], which more accurately reflects the actual situation through the offset placement of the COM. The torso is treated as a rigid body with both mass and rotational inertia. Yu and Iida [135] further enhanced the model by incorporating the influence of foot-end mass [Fig. 5 (1c)], which adds an additional mass block below the original one to represent the foot-end mass. The rotation of the third mass block connected to the upper mass block realizes the model to jump, consistent with the mass-spring system motion.

In addition to the model improvement related to mass, researchers have found through studying the running behavior of biological organisms that energy production and consumption are regulated through muscle actuation during the process of biological movement [136,137]. However, the traditional SLIP model is energetically conservative, neglecting energy loss due to collisions and viscous damping. As a result, it fails to fully capture the robustness of SLRs, limiting their performance in unstructured terrain or high-speed motion scenarios. Consequently, Uyanık et al. [138] proposed a dissipative SLIP (D-SLIP) model incorporating passive and compliant damping to the model [Fig. 5 (1d)]. This extended model successfully reflects energy loss and demonstrates its effectiveness in capturing dissipative behaviors [139]. Additionally, the legs inject energy into the system to compensate for the energy loss during contact with the ground. Secer and Saranli [140] introduced a series drive with a piston structure to form Active-SLIP, which actively injects energy into the legs during movement, aligning more closely with biological movement [Fig. 5 (1e)]. Further advancing this concept, Hamzaçebi and Morgül [141] proposed a multi-actuated D-SLIP model [Fig. 5 (1f)], with a linear actuator attached serially to the leg spring and a rotary actuator attached to hip. The model allows for the derivation of analytical solutions for stance dynamics, enabling robust motion control in rugged terrain.

Beyond standard structured ground scenarios, the applications of legged robots in special environments also imposes additional challenges [142]. Calisti’s [143,144] underwater SLIP (U-SLIP) model incorporates specific hydrodynamic contributions, such as drag, buoyancy, and added mass [Fig. 5 (1g)]. By accounting for these factors, the U-SLIP model facilitates self-stabilizing jumping behavior in aquatic environments, thereby extending the applicability of legged locomotion models to submerged conditions.

Traditional SLIP models fail to accurately capture the locomotion dynamics of robots like RHex, which utilize circular elastic elements or wheels as foot-end instead of traditional foot tips. To address this limitation, Huang et al. [145] proposed a rolling SLIP (R-SLIP) as shown in Fig. 5 (1h), modifying the original spring-mass system by introducing a circular rolling foot coupled with a torsional spring. This adaptation endows the model with intrinsic rolling dynamics and variable stiffness. This method has been widely applied in the modeling of circular SLRs [146,147]. Similarly, wheel-legged robots combine the characteristics of wheeled and legged robots, and researchers have proposed the 2-DoF SLIP model tailored for wheel-legged robots by incorporating driven wheels at the bottom end of the conventional SLIP model. Chen et al. [148] established the wheeled-SLIP (W-SLIP) model [Fig. 5 (1i)], which only considers the mass of the driving wheel and ignores the moment of inertia. This simplification reduces the complexity of the overall dynamics while capturing the underactuated characteristics of the wheeled robot. Based on the W-SLIP model, the wheel-legged SLIP (W-LS-LIP) model [Fig. 5 (1j)] further considers the relationship between the floating base, leg mechanism, and driving wheels of the wheel-legged robot [149], thereby enabling the robot to adapt to more complex and unstructured terrains.

To enhance robot balance under impacts from different directions, further research have expanded the 2-DoF SLIP model to the 3-DoF. Seipel and Holmes [150] investigated the stability of single leg operation using Poincaré analysis through the 3-DoF SLIP model [Fig. 5 (1k)]. This approach allowed for a deeper understanding of dynamic stability in legged locomotion. Han et al. [151] proposed a 3D-SLIP model [Fig. 5 (1l)] consisting of a concentrated mass body and equivalent leg springs connected via a 2-DoF rotating hinge, which enables multi-directional leg movement through telescopic spring action.

#### Articulated model

The SLIP model greatly simplifies the kinematics of SLRs, effectively reducing the complexity of modeling. However, its abstraction omits critical aspects such as joint articulation, actuator dynamics, and mechanical constraints, making it inadequate for achieving the necessary control accuracy in complex environments or when performing fine operations. In contrast, articulated models explicitly incorporate the robot’s structural configuration and joint DoFs, enabling more accurate representation of intricate movements and task-specific control requirements. Based on the inclusion of elastic elements, articulated models can be categorized into springless articulated models and springy articulated models.

The springless articulated model exclusively considers the rigid joints and links of the mechanical structure. A representative example of a springless articulated [Fig. 5 (2a)] model is presented by Berkemeier and Fearing [152], in which the mass of each link is concentrated at the joints. This modeling approach is employed to investigate the influence of link mass distribution on the overall dynamic behavior of the robotic system. Subsequently, considering the frictional effects arising from fitting errors and surface roughness, Roozing [153] established a model [Fig. 5 (2b)] to reflect the friction and energy loss during motion by dampers.

One of the primary challenges in constructing SLR models is the dynamic nature of the moving coordinate system. Therefore, Chen et al. [154] connected the leg system to a massless 2-link virtual manipulator [Fig. 5 (2c)]. This abstraction simplifies the original limb structure into a serial kinematic chain comprising 6 links and 7 joints, thereby facilitating model-based motion planning and control. Building upon the concept of decoupling [155], Zhang et al. [156] further proposed a virtual decoupling decomposition model [Fig. 5 (2d)], which transforms the original closed-chain configuration formed by hydraulic cylinders into an equivalent open-chain representation. This approach enables high-precision dynamic modeling of hydraulic closed-loop mechanisms [157], without imposing significant computational burden.

The springy articulated model incorporates elastic elements, enabling the system to actively adapt to varying terrains that simulate biological organisms.

Schwind et al. [158,159] proposed 2 massless elastic knee joint rotating leg models. The first “Spring Loaded Small Knee” model [Fig. 5 (2e)] features massless ankle and knee joints, with passive ankle and knee joints mounted on an active rotating hip joint connected to the main body. This model effectively captures the elastic behavior of a single leg in biology. Another model is called “Ankle–Knee–Hip” [Fig. 5 (2f)], which incorporates rotating ankles, knees, hip joints, and massless toes, drawing inspiration from the locomotion of humans and kangaroos. The inclusion of the rotary actuators facilitates optimal landing postures and emulates virtual spring stiffness.

To more accurately capture the kinematic and dynamic characteristics of SLRs in diverse application scenarios, researchers have developed a series of specialized articulated models tailored to specific environments and mechanical configurations. Astolfi et al. [160] improved an articulated model [Fig. 5 (2g)] that describes the dynamics of a point mass immersed in water. This model represents the leg as a 2-segment articulated structure and incorporates the effects of underwater currents and buoyancy, enabling dynamic analysis and control in aquatic environments. To address the unique biomechanical behavior of curved feet during running, such as rolling contact motion, variations in effective leg stiffness and rest length, and compliant vaulting over the toe during stance, Jun and Clark [161] proposed a novel torque-driven reduced-order dynamic model called torque-driven and damped half-circle-leg, effectively capturing the dynamic properties of curved-foot locomotion. The articulated-wheel inverted pendulum model features a multilink articulated structure mounted above a wheel, which effectively captures the high nonlinearity, underactuation, and inherent instability commonly observed in legged systems [162]. The characteristics endow the model with the potential to adapt its joint configurations in response to varying task demands across different environments.

Although articulated models provide a more structurally faithful abstraction of physical robotic systems, their accuracy in real-world scenarios is often constrained by various nonidealities, including mechanical assembly tolerances, uncertainties in ground contact conditions, and limitations in sensor resolution. These discrepancies can introduce marked divergence between model-based simulations and empirical system performance. Therefore, developing application-specific models tailored to particular environments and operational conditions remains a critical challenge in current modeling efforts.

### Control strategy design

Legged robots possess the capability to address the challenges posed by high-dimensional systems, including floating-base dynamics and redundant DoFs [163,164]. Furthermore, the complexities introduced by whole-body multicontact interactions with unknown environments further complicate the development of effective control strategies. The control framework for legged robots generally comprises 2 primary components: the upper-level motion controller and the lower-level joint controller. Considering the relatively mature force/position control of joints, this paper focuses on the design of upper level planning and control strategies, with the goal of generating the target position, velocity trajectory, or ground reaction force to achieve the locomotion behaviors.

**Fig. 7.**
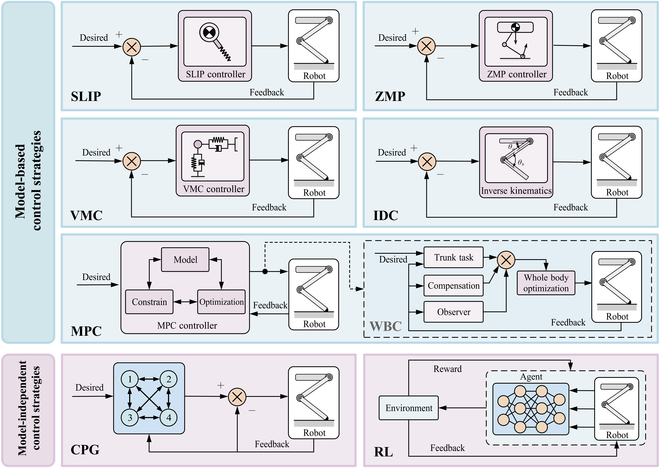
Overview of model-based and model-free control strategies for SLRs.

Control strategies for SLRs can be broadly classified according to their reliance on explicit system models. Model-based control strategies leverage system dynamics for precise control, whereas model-free control strategies operate without requiring an explicit model of the robot’s dynamics, relying instead on data-driven approaches or heuristic methods to generate control inputs, as shown in Fig. 7.

#### Model-based control strategy

##### SLIP control strategy

The SLIP-based control strategy simplifies the structure into a SLIP for motion planning, which describes the transformation between kinetic and potential energy throughout the motion cycle [165,166]. By leveraging this model, the robot can achieve jumping behavior through coordinated transitions between the stance and flight phases [167].

**Fig. 8.**
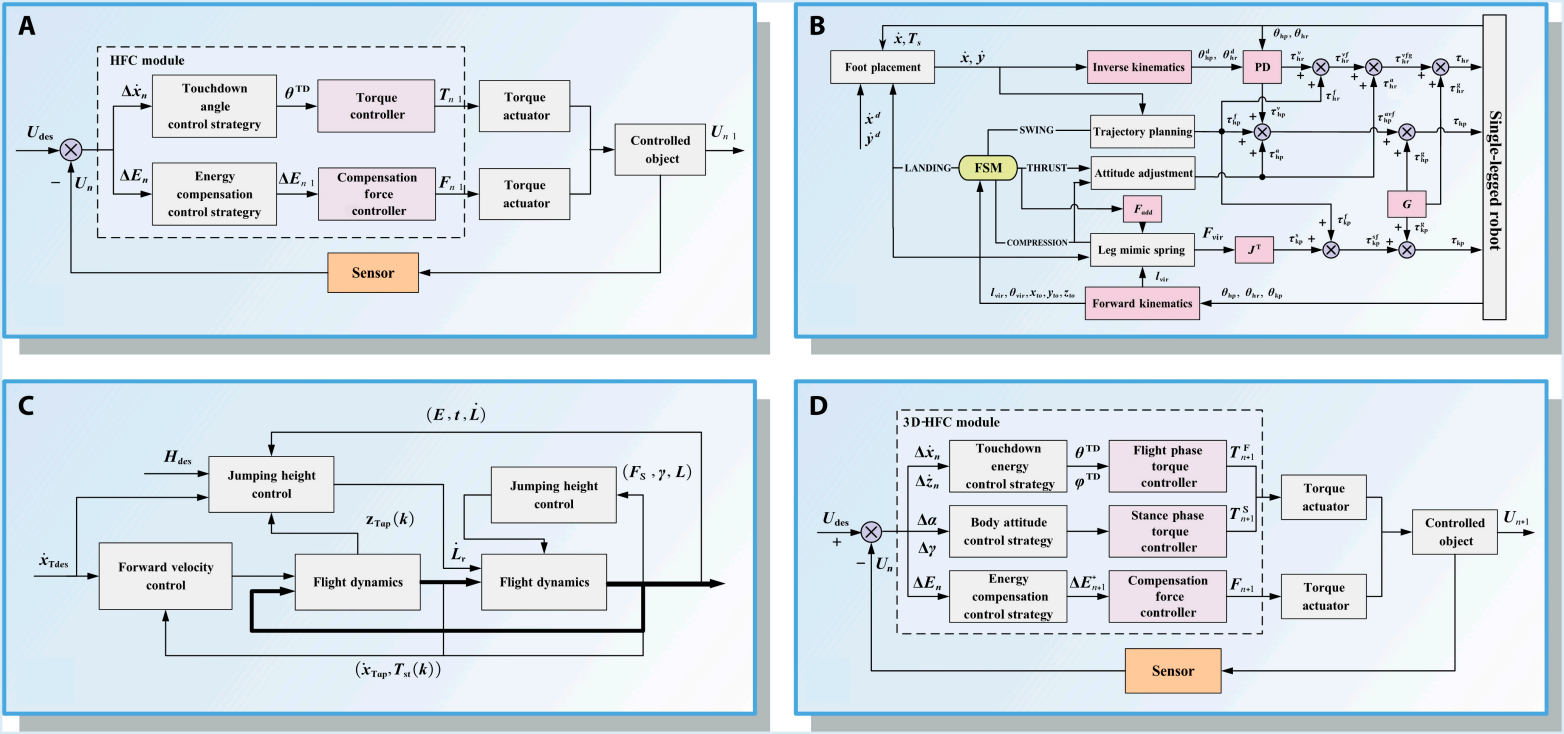
Decoupled SLIP control strategy frameworks. (A) HFC method. (B) Obstacle-clearing SLIP control method. (C) Extended SLIP control method. (D) 3D-HFC method.

Raibert [18] proposed the first SLIP-based control strategy known as the “three-part control”, which decomposes the hopping control process into 3 distinct modules: forward motion control based on foot placement planning, hopping height control via energy modulation, and posture adjustment control using a posture feedback loop. Building upon the original 3-part control method, Cherouvim and Papadopoulos [168] proposed a simplified method that employs hip torque to simultaneously regulate both forward velocity and height, thereby reducing the complexity of motion planning to a certain extent. Furthermore, Han et al. [169] proposed a feasible hybrid feedback control (HFC) strategy, as illustrated in Fig. 8A, which consists of touchdown angle control and energy compensation. By leveraging hybrid feedback from the flight apex state, the strategy predicts and adjusts the touchdown angle of the current cycle while determining the required energy input for the next cycle. The HFC strategy can realize rapid and stable control across various terrain conditions. Huang and Zhang [170] presented an obstacle-clearing control method (Fig. 8B) for a bio-inspired SLR that combines the SLIP model with Bézier curve-based foot trajectory planning. The finite state machine module is responsible for coordinating the execution of actions in different states, enabling the robot to continuously clear obstacles of varying heights in simulated experiments. With advances in SLIP models, researchers have also conducted new control strategies for special environments. Luo et al. [171] proposed an improved control strategy based on the extended SLIP model, as shown in Fig. 8C, which consists of posture stabilization control, hopping height control, and forward velocity control. During the stance phase, posture stabilization is achieved by incorporating an approximate virtual pendulum posture control with fixed point to ensure smooth torso motion. During the flight phase, an improved foot placement method is introduced to ensure precise forward velocity tracking. Kang et al. [172] introduced a periodic running control strategy based on a novel quasi-linearized SLIP (QL-SLIP) model, which integrates additional forces in both radial and angular directions; the strategy enhances stability across different running speeds in the presence of external disturbances. Han et al. [151] introduced a 3D-HFC control method (Fig. 8D) that comprises 3 core modules: the touchdown angle control module regulating forward motion, the attitude angle control module mitigating accumulated deviations in body posture induced by the nonnegligible leg mass, and the energy compensation control module counteracting damping effects of the leg spring and energy loss during ground contact.

Nonetheless, decoupled SLIP control strategies exhibit several inherent limitations. First, the conventional SLIP model assumes ideal energy conservation, which restricts its applicability to hopping on uneven terrain. Second, the SLIP model’s highly nonlinear and coupled dynamic equations prevent the derivation of an exact analytical expression for stance-phase dynamics, thereby complicating precise characterization of the hopping process. Finally, the model’s inherent simplifications neglect certain limb details, further complicating precise control.

##### Zero moment point control strategy

Zero moment point (ZMP) is defined as the point on the support surface where the resultant moment caused by inertial and gravitational forces becomes zero. If the ZMP remains within the foot support region in contact with the ground, the robot is considered at a balanced and stable state. Leveraging this stability criterion, the motion trajectory of the robot can be strategically planned [173,174].

**Fig. 9.**
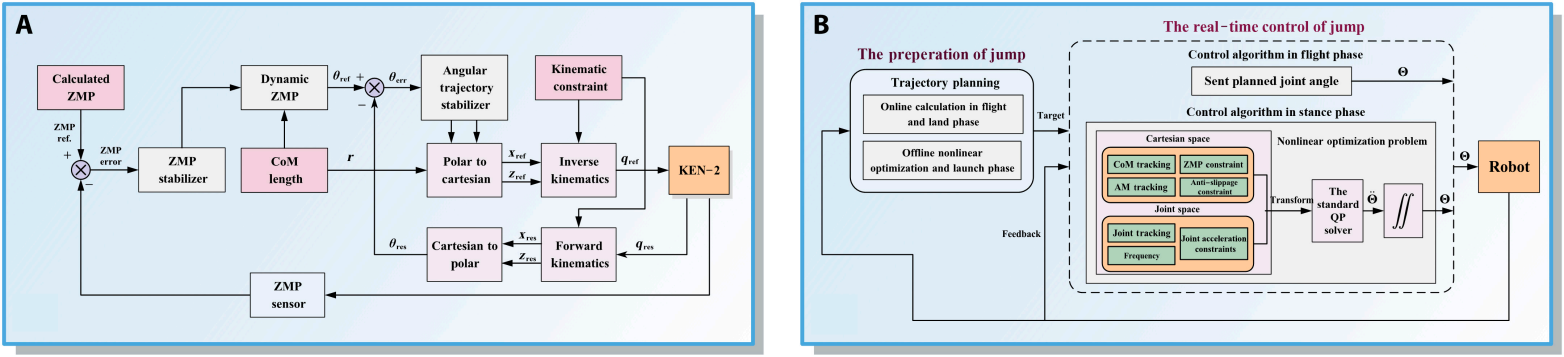
Zero moment point control strategy frameworks. (A) ZMP-based global dynamic balance control method. (B) QP optimization control method.

The Toyota research group applied an online ZMP-based analytical method to humanoid robot hopping experiments [175]. By discretizing the ZMP equation, they numerically computed the trajectory for hopping, thereby lowering the demands on the actuators. Ugurlu and Kawamura [176,177] proposed a ZMP-based global dynamic balance control strategy (Fig. 9A) that enables stable hopping motion without relying on angular momentum information. During the stance phase, the ZMP is selected as a reference to compute the CoM trajectory, which is then mapped to joint space and considered as inputs for each servo controller. During the flight phase, the trajectory is conducted based on the conservation of linear momentum. Subsequent research further extended the ZMP-based method to humanoid robots in Ref. [178], enabling real-time generation of jogging and hopping trajectories. Jiang et al. [179] proposed a control scheme for bipedal robots, where the jumping pattern was precomputed to maximize jump height, achieving a recorded height of 0.477 m. Tian et al. [180] introduced a quadratic programming-based optimization framework (Fig. 9B), which simultaneously considers ZMP, angular acceleration limits, and slip prevention. Trajectory generation involves discretizing the continuous trajectory and transforming the problem into a nonlinear optimization task. Real-time control integrates over-constrained objectives along with constraints. The robot successfully achieved a 16.4-cm jump height while ensuring all constraints are satisfied.

However, the ZMP control strategies require the foot of SLR to maintain a support polygon configuration, rendering them inapplicable to point-contact single-leg scenarios [181]. Moreover, the application of ZMP-based control is constrained by its stringent stability requirements and heavy reliance on ground flatness, limiting the effectiveness to accommodate rapid or complex motion tasks [182].

##### Virtual model control strategy

Pratt et al. [183] proposed an intuitive leg motion control strategy named virtual model control (VMC), which places virtual components (such as springs, dampers, and dashboards) between contact points. By analyzing the mechanical properties of the virtual components to compute virtual forces and moments, they are applied to the controlled object to achieve the desired motion characteristics. Once the virtual model is established, the original dynamic model can be disregarded, substantially simplifying the modeling and control process. In addition, VMC can be readily integrated with advanced control schemes, such as adaptive control, neural network, and stiffness control, enabling it to adapt to various dynamic environments [184,185].

**Fig. 10.**
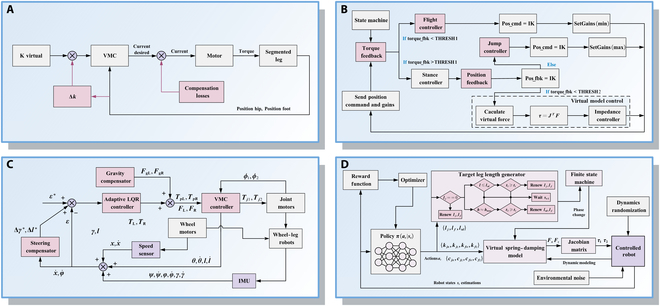
Virtual model control strategy frameworks. (A) MARCO-Hopper II control method. (B) Hopping virtual model control method. (C) Parameter adaptive VMC method. (D) Adaptive learning algorithm VMC method.

Oehlke et al. [186] employed VMC as a feedback control method to stabilize the MARCO-Hopper II (Fig. 10A), adapting the required knee torque during the hopping process by considering the distance between the hip and foot, the actual length of the virtual spring, and the virtual stiffness. Compared to the feed-forward method, this approach demonstrated superior perturbation recovery and locomotion adaptability. He et al. [187] designed a control method based on VMC state switching and control parameter optimization. They utilized the VMC law with constant virtual stiffness, damping, and initial leg length to maintain the stability and compliance of the robot, enabling highly dynamic movement during jumping and landing. Kalouche [188] achieved compliant monopod jumping through the VMC method (Fig. 10B), where high-bandwidth motors emulate virtual spring and damper components to replicate the forces generated by mechanical springs and dampers, thereby enhancing the flexibility of the overall leg. Yang et al. [189] proposed a parameter adaptive strategy for the linear quadratic regulator and response optimization compensators shown in Fig. 10C. The mapping between the force/torque of the virtual leg and the joint torques is obtained by solving the forward kinematics equation and applying the virtual work principle, simplifying the dynamics model of parallel-legged structures. Inspired by bionics theory, Sun et al. [190] proposed a perception-driven highly dynamic jump adaptive learning algorithm by combining learning algorithms with the VMC method (Fig. 10D). This method adjusts the 2-DoF motion of a single leg by controlling the spring and damper optimal parameters optimized via learning, achieving continuous and ideal vertical jumping motion after minimal training.

The stiffness and damping of the virtual spring function similarly to the proportional and derivative gains in proportional-integral-derivative (PID) control. When the robot deviates markedly from its desired posture, a large torque is needed, thereby compromising both the robot’s stability and the duration of sustained locomotion. Another major limitation of VMC is that virtual components cannot fully capture the dynamic characteristics of the system. Inertia effects and sensor noise can further exacerbate this limitation. Moreover, the VMC method primarily focuses on control at the current moment, lacking predictive capabilities for future motion or environmental variations [191].

##### Inverse dynamics control strategy

The inverse dynamics-based control (IDC) strategy achieves control by solving the robot’s precise dynamic model equations in a reverse manner. The core concept of this approach is to directly compute the required joint torques based on the desired joint motion trajectory and the robot’s dynamic model. Through this process, the control system can precisely regulate the robot’s motion state, ensuring that it follows the predefined trajectory accurately.

Naik and Mehrandezh [192] implemented a PID controller based on inverse kinematics to achieve jumping in the SLR. The PID feedback control was used to adjust the duty cycle of the PWM signal, enabling the robot to reach a specific jumping height and maintain a constant jumping height. Zhang et al. [193] proposed a single-legged unit inverse dynamics model based on the Lagrangian to reduce landing impact, simulating human knee bending and cushioning movements. Zhou et al. [194] proposed an inverse dynamics controller combining partial feedback linearization and sliding mode control, fully accounting for nonlinear couplings in wheel-legged robots. It enables accurate configuration transformation with minimal floor drift. An optimization-based planner further enhances task precision, offering improved stability and adaptability. Zhang et al. [156] proposed a model-based virtual decoupling control framework to manage the closed-chain hydraulic structure, which includes 3 VDC-based controllers designed separately for the stance and swing phases of the single-leg motion, which demonstrates superior position trajectory tracking performance and jumping capability.

The implementation of the IDC strategy necessitates an accurate dynamic model. However, obtaining an accurate dynamic representation in dynamic and real-time environments is often difficult and the presence of sensor noise further increases system complexity. Moreover, deriving inverse dynamics equations involves intensive computation, imposing a substantial computational burdens on the system.

##### Model predictive control strategy

Model predictive control (MPC) is an optimal control strategy that computes the optimal control inputs based on the system model by considering the current state and predicting its future behavior [195,196]. By forecasting future state trajectories and optimizing control inputs, MPC adjusts the control strategy at each time step in response to the current system state, enabling the robot to adapt to dynamically changing environments and task requirements [197,198].

MPC integrates environmental constraints and dynamic goals directly into the cost function, enabling the robot to optimize motion performance. Rutschmann et al. [199] proposed an MPC strategy for robust jumping foothold planning. By incorporating the squared foothold error at each step and the continuity of apex velocity into the cost function, the landing error during hopping is limited to within 20% of the leg length while considerably reducing computational costs. To enhance the ability of SLRs to traverse obstacles, Albracht et al. [200] employed a mixed-integer motion planning method in the model predictive parkour control strategy for parkour-style jumping. The strategy minimizes the total duration of all flight phases while optimizing the best trajectory in dynamically changing obstacle environments with physical collision considerations. Moreover, Le Cleach et al. [201] extended the applicability of linear MPC to contact-rich scenarios by proposing a contact-implicit formulation based on bilevel optimization. This approach improves trajectory adaptability and computational robustness during dynamic transitions, such as hopping and stance switching, on uneven or shifting terrain. Collectively, these studies underscore MPC’s capability to enable collision-free motion planning and real-time adaptability.

**Fig. 11.**
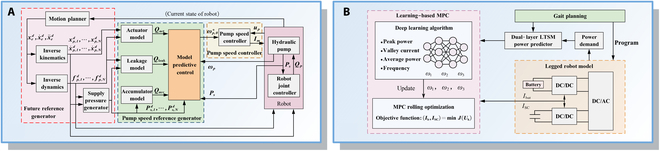
Model predictive control strategy frameworks. (A) Energy-efficient hydraulic pump MPC method. (B) Learning-based MPC energy management method.

In addition to enhancing motion performance, MPC also facilitates energy-efficient control by incorporating energy consumption into the cost function. Cho et al. [202] addressed the problems of energy efficiency and heat dissipation in hydraulic robots by introducing an MPC-based strategy (Fig. 11B). This method formulates the energy consumption of each component as part of the optimization objective and employs nonlinear MPC to generate the pump speed reference trajectory, thereby achieving energy-efficient and stable operation of the hydraulic robot. Furthermore, Shu et al. [203] proposed a learning-based MPC strategy for energy management in legged robots equipped with a hybrid battery–supercapacitor energy storage system. The controller integrates a power prediction module and a learning-based adaptive weighted MPC controller, which uses a dual-layer long short-term memory network to predict load power demand. Compared to a conventional MPC controller, this method reduces battery capacity degradation by 12.7% during locomotion.

Due to the highly nonlinear and complex dynamics of SLRs, implementing MPC for precise control necessitates substantial real-time computational resources. To mitigate this challenge, optimization solvers such as the interior-point method [204] and the active-set method [205] have been employed to significantly reduce the computational cost of online MPC. Notably, open-source solvers [206,207] have been integrated, enhancing the feasibility of MPC in highly dynamic and complex environments.

Meanwhile, the control frequency of MPC is constrained by the computational cost, which is insufficient to meet the demands of complex, high-frequency motions in legged robots. As a result, whole-body control (WBC) is frequently employed as a low-level controller to execute motion trajectories generated by MPC. The core principle of WBC is to manage multiple tasks with various constraints by assigning priorities, enabling efficient task allocation in robotic systems [208,209]. However, when a robot has insufficient degrees of actuation, WBC may fail to complete the task as intended. Such limitations are commonly encountered in SLRs. In contrast, WBC is more commonly applied in the control of MLRs, where greater actuation redundancy provides greater flexibility in task execution. When environmental characteristics are imprecisely defined or long-term control is required, the robustness of WBC deteriorates, resulting in suboptimal control performance.

#### Model-free control strategy

Although model-based control strategies have demonstrated experimental success, there are still 2 shortcomings. First, the accuracy of the model has inherent limitations and cannot perfectly reproduce all the characteristics of actual SLR systems, such as physical parameters and response speed. Second, when confronted with complex nonlinear structures, the process of solving the model equations often becomes very difficult, especially in highly dynamic and unpredictable systems.

In contrast, model-free control strategies do not rely on the precise system model and directly control based on input–output data, thus offering greater flexibility and robustness in adapting to dynamic changes and environmental uncertainties.

##### Central pattern generator control strategy

The central pattern generator (CPG)-based control strategy aims to replicate the rhythmic movements of organisms, which are generated by the autonomous activity of neural centers located in the spinal cord [210]. Multiple layers of neuron pools are coupled with oscillators, forming a self-organizing network through complex interconnections [211]. By leveraging CPG principles, robotic systems can mimic natural animal locomotion, enabling more natural, flexible, and efficient motion control [212].

Yoshida et al. [213] implemented a CPG-based feedback controller to regulate the control input current of the internal electric actuator. The parameters of the CPG are optimized through genetic algorithms to minimize the error between the expected jump height and the actual height, enabling controllable continuous jumping motion. Zhang et al. [214] proposed a motion control method based on the gradient CPG (GD-CPG). The periodic signals generated by the neural network serve as the drive signals for each thigh joint of the legged robot, and then are transformed into the driving signals by the thigh-knee mapping and knee-ankle mapping function. The GD-CPG realizes control of the entire leg, which reduces computational complexity and improves efficiency. Schmidt et al. [215] tested 3 control methods on a biomimetic robotic leg: the reflex representing control, the CPG control strategy with feedback, and the pure CPG strategy. The experiments showed that the CPG controller with feedback exhibited the fastest recovery time after a fall disturbance.

The performance of the CPG-based control strategy heavily depends on the proper parameter setting. Appropriate neuron design and interconnection can significantly enhance the responsiveness and performance of the system. However, establishing the relationship between parameters and output results, such as the design of neuron structure and connection methods, requires extensive experimentation and resources.

##### Reinforcement learning control strategy

Reinforcement learning (RL) is a learning process that interacts with the environment through trial and error, with the primary objective of learning optimal actions in different contexts to maximize cumulative rewards, thereby achieving efficient and stable motion control in changing contexts [216,217].

**Fig. 12.**
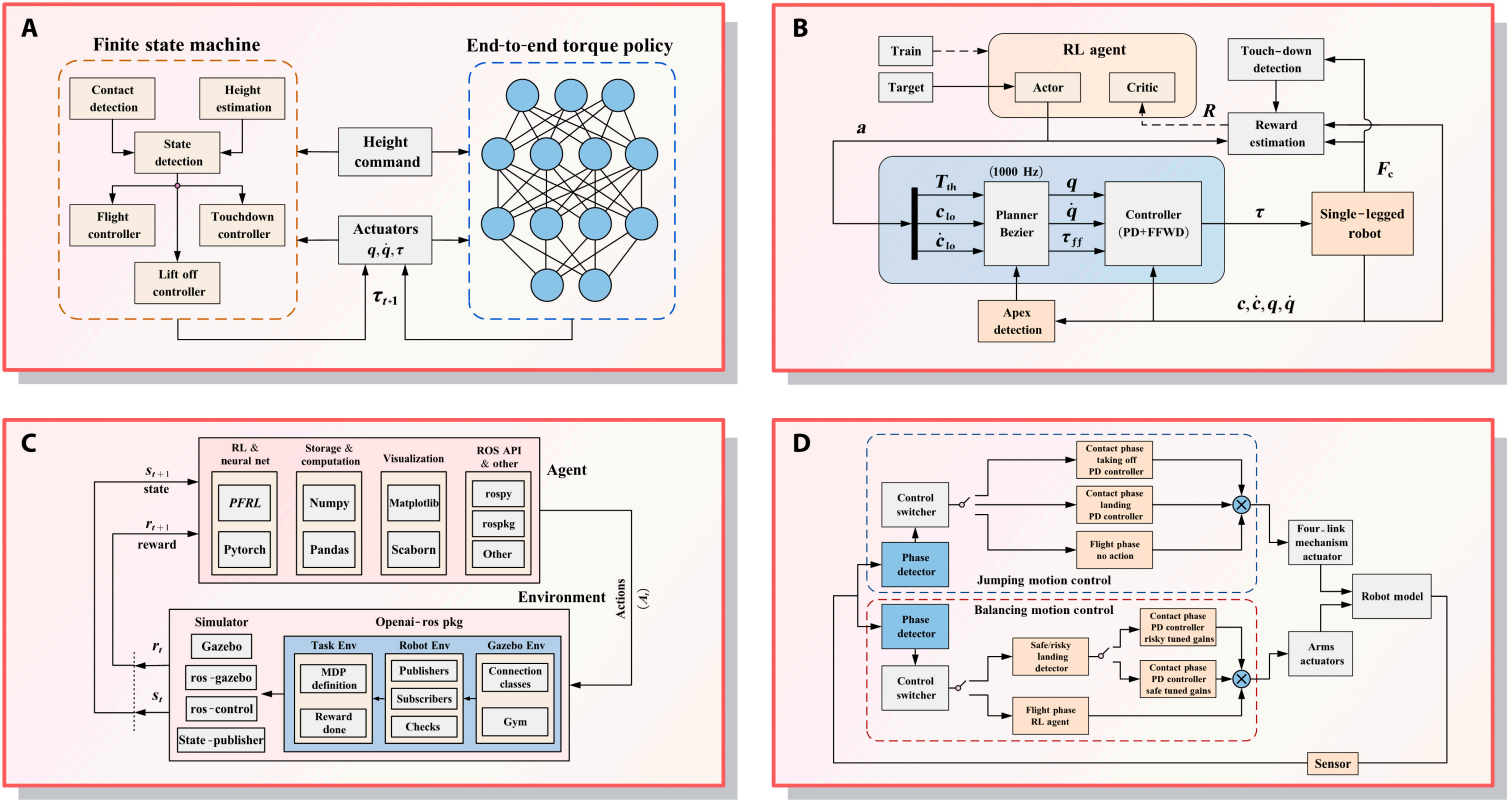
Reinforcement learning control strategy frameworks. (A) End-to-end RL-based continuous jumping control method. (B) Guided reinforcement learning control method. (C) Four RL algorithms control method. (D) Hybrid DDPG control method.

Compared with traditional control strategies, RL-based control can effectively mitigate challenges associated with model dependency and computational complexity, offering researchers novel solutions for handling uncertainty and complex tasks [218,219]. Soni et al. [220] proposed an end-to-end RL based torque continuous jumping control method (Fig. 12A) that autonomously calculates optimal joint actuator angles and torques by directly inputting the desired jump height. Bussola et al. [221] proposed a guided RL control strategy in Fig. 12B, in which the agent is responsible for generating Bezier curves based on the target, and a planner maps the curves to joint movements. Rewards computed at the end of each episode are fed back to the RL agent. Compared with end-to-end methods, it achieves comparable or superior performance while maintaining real-time computational efficiency.

To expand the behavioral repertoire of SLRs, Moslemi et al. [222] trained an SLR equipped with active toe joints using 4 RL algorithms, as illustrated in Fig. 12C. By leveraging a reward function design, the system achieved complex maneuvers such as squat jumps and knee-hugging jumps without the need for predefined trajectories. Similarly, Choe et al. [223] proposed a 3-DoF hopping robot with an RL-based controller to address challenges in high-torque and agile motion control. The RL controller, trained with barrier-based rewards and closed-loop dynamics simulation, enables stable repetitive hopping, front flipping, and push recovery.

In addition to task-specific designs, another line of research has focused on enhancing performance by modifying and combining RL algorithms. These hybrid or extended frameworks aim to improve convergence stability, sample efficiency, and robustness of control. Hoseinifard and Sadedel [224] proposed a hybrid control strategy that combines the deep deterministic policy gradient (DDPG) algorithm with a feedback controller (Fig. 12D). The algorithm governs the coordinated movement of the arms and lower body in 3D space during the flight phase. By leveraging this hybrid strategy, the robustness is enhanced in both jumping execution and balance maintenance. Kim et al. [225] proposed an extended maximum actor–critic framework tailored for SLR jumping, which integrates policy gradient techniques to enhance the training stability and incorporates prioritized hindsight experience replay to effectively utilize sensory feedback for improved control robustness. This method achieved a 27.67% improvement in average compared to the baseline TD3 algorithm.

The application of RL methods presents marked challenges, primarily due to the high-dimensional state and action spaces required for effective training. For instance, the training process in complex terrain necessitates not only the evaluation of torso posture but also the real-time assessment of foot position, orientation, and velocity, where the dimensionality of the states and behaviors involved in this process can reach millions.

Moreover, transferring RL policies trained in simulation to real-world robotic platforms has become a critical and unresolved challenge. While virtual environments enable efficient training with diverse terrains and idealized models, the transition to real-world deployment is often hindered by discrepancies including sensor noise, actuator delays, and contact uncertainties [226,227]. Direct deployment of policies trained in simulation often leads to great performance degradation or even complete failure in real-world scenarios. To address this issue, researchers have proposed the “Sim-to-Real” transfer techniques, including domain randomization, the development of high-fidelity physics-based simulators, and the incorporation of imitation learning, which enables training the model in a quasi-realistic environment and then adaptively adjusting it to run in a real environment while maintaining robust performance and stability [228].

#### Summaries of the SLR control strategy

As outlined in the preceding sections, substantial progress has been made in the development of control strategies for SLRs. While existing control strategies demonstrate unique advantages in terms of model formulation, control precision, adaptability, and computational feasibility, they also entail distinct limitations and trade-offs, particularly in real-world deployment. The comparative analysis of representative SLR control strategies are presented in Table 7. This comparison is intended to offer theoretical insight and practical guidance for selecting appropriate control frameworks under varying task requirements, robot configurations, and operational contexts.

**Table 7.** Comparative analysis of representative control strategies for SLRs

Control strategy		Advantages	Limitations
Model-based control strategy	SLIP strategy	1. Conceptually simple and analytically tractable 2. Effectively models CoM dynamics observed in biological locomotion 3. Suitable for rapid prototyping and simulation-based analysis	1. Lacks joint-level dynamic fidelity 2. Inaccurate modeling of energy consumption and control precision
	ZMP strategy	1. Provides high stability in quasi-static gait generation 2. Well-established in bipedal and humanoid robot control frameworks	1. Inadequate for dynamic or agile locomotion 2. Neglects limb compliance and nonlinear ground interactions
	VMC strategy	1. Physics-inspired and intuitive to implement 2. Enables flexible realization of diverse locomotion behaviors 3. Easily integrates with adaptive control and learning methods	1. Sensitive to parameter tuning 2. Limited precision due to virtual approximation 3. Assumes specific structural or behavioral characteristics
	IDC strategy	1. Provides high spatial accuracy for end-effector or limb trajectory tracking 2. Integrates well with preplanned motion sequences	1. Requires accurate dynamic model, often difficult to obtain in real time 2. Sensitive to sensor noise, reducing robustness 3. Inverse dynamics computation is intensive, increasing real-time computational burden
	MPC strategy	1. High control accuracy with optimal predictive capabilities 2. Capable of handling multivariable systems and complex task constraints 3. Allows incorporation of additional objectives while satisfying primary task goals	1. Computationally demanding, challenging for real-time implementation on embedded systems 2. Requires accurate system modeling
Model-free control Strategy	CPG strategy	1. Bio-inspired, naturally produces smooth, rhythmic motion patterns 2. Facilitates inter-limb coordination and stable gait generation	1. Limited adaptability to non-periodic or task-specific motions 2. Requires extensive parameter tuning for different robots or environments
	RL strategy	1. Learns complex control policies through interaction 2. Highly adaptable to varying environments and uncertainties 3. Well-suited for high-DoF and nonlinear systems	1. High training cost and data inefficiency 2. Poor interpretability compared to model-based methods 3. Requires substantial engineering effort to transfer trained policies to real-world hardware

## Future Research and Challenges

**Fig. 13.**
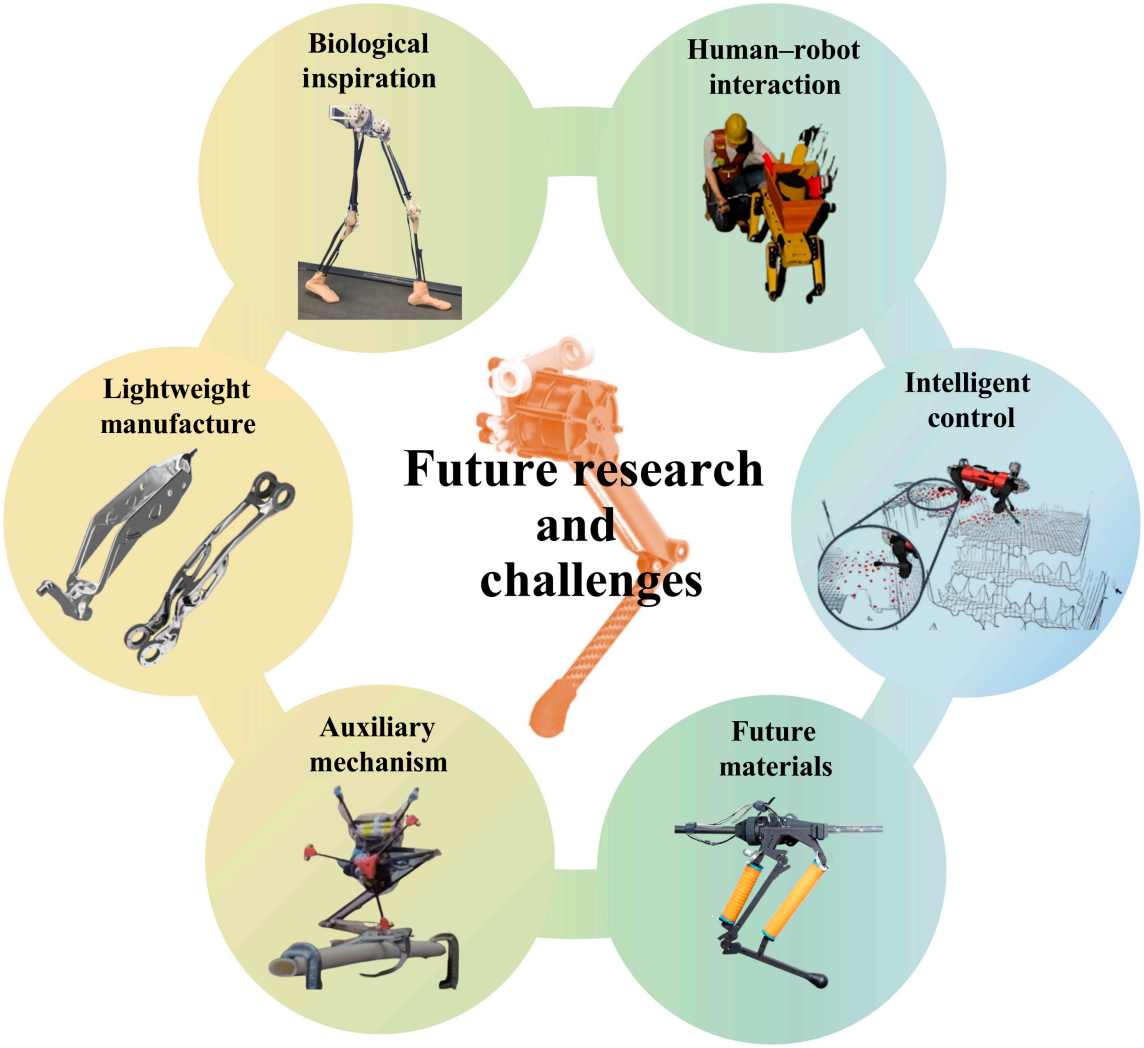
Future research directions and challenges for SLRs.

Based on the reviewed aspects in this paper, the past 50 years have elevated the field of SLRs to a new level. The developments in materials, processes, and control theories have opened new avenues for fundamental research of SLRs. This section identifies future research directions and challenges as shown in Fig. 13.

### Biological inspiration

Legged robots are inherently inspired by the principles of bionics, mimicking the fine structure and motion characteristics of organisms to optimize artificial systems [229,230]. Recent advancements have demonstrated excellent results compared to traditional configurations, such as the bionic ostrich robot in Ref. [231], the insect-inspired robot in Ref. [232], and the robot referencing human knee femur in Ref. [233]. However, biological systems exhibit nonlinear, heterogeneous, and multifunctional structural features, which are challenging to replicate by manufacturing techniques and conventional engineering materials. Moreover, abstracting biological functions into simplified mechanical counterparts involves intricate trade-offs between biological fidelity and engineering feasibility.

### Lightweight manufacture

Weight remains a critical constraint in the development of mobile robots, which directly determines their energy efficiency and operational endurance of robots. To address this challenge, reducing the structural mass of robots through advanced techniques such as topology optimization [234,235], generative design [236], and multi-material additive manufacturing [237,238] has emerged as one of the core directions for future research. Nonetheless, the practical implementation of these methods continues to face substantial challenges, particularly in ensuring mechanical robustness under dynamic loading conditions and navigating the inherent trade-offs among structural performance, manufacturability, and cost-efficiency.

### Auxiliary structure

Purely legged structures often constrain the operational scope of robots, especially in complex environments. To enhance mobility and versatility, the development of auxiliary mechanisms is also crucial, such as the application of the simple passive gripper in Ref. [239], the introduction of reaction wheels in Ref. [240], the use of tails in Ref. [241], and the integration of flight devices in Ref. [43]. The integration of auxiliary structures may substantially alter the robot’s COM and dynamic behavior, thereby increasing the complexity of motion control and introducing undesired attitude disturbances during locomotion. Moreover, such augmentations often result in mechanical layout conflicts, increased wiring complexity, and thermal management issues.

### Future materials

The emergence of advanced materials has opened new possibilities for improving the performance of robots [242,243]. By utilizing materials with superior energy storage capabilities compared to traditional steel springs, the rubber–carbon fiber composite jumping robot [244], the novel insect-scale shape memory alloy jumper [245], and the light-driven soft jumping robot [246] exhibit remarkable jumping capabilities. However, the practical implementation of future materials still encounters considerable technical challenges, including fabrication complexity, long-term durability, and seamless integration with conventional actuation mechanisms.

### Intelligent control

Legged robots function as complex multi-sensory systems, requiring real-time data processing and precise actuator control to maintain stability and adaptability. Recent studies have explored integrating RL techniques with privileged learning to enable mobility-aware local navigation and large-scale path planning in Ref. [247], and the fully learned control enables robots to handle challenging scenarios in Ref. [248]. The study of dynamic control for legged robots in complex environments is undoubtedly tricky. Achieving optimal performance in complex environments requires overcoming critical challenges such as limited computational resources, real-time responsiveness, prolonged training durations, and the sim-to-real transfer gap.

### Human–robot interaction

With the continuous advancement of legged robots and the increasing potential for full autonomy, their integration into everyday human environments is becoming progressively feasible, including ensuring human safety in shared workspaces such as construction sites [249,250], enabling semantic understanding and precise navigation in unstructured environments [251–253], and maintaining human-centered control paradigms in applications like rehabilitation exoskeletons [254,255]. Ensuring physical safety, transparency, and personal privacy remains challenging in human–robot interaction. These issues underscore the urgent need to investigate the ethical implications and safety considerations associated with the real-world deployment of legged robots [256].

## Conclusions

This article on SLRs comprehensively reviews the research progress of the mechanism design and control strategy of SLRs. The following viewpoints are obtained to summarize their development process.

1. The telescopic configuration has been widely recognized and explored in the early research of SLRs due to the simple structure and simple jumping planning method. However, due to the limited DoF and restricted space for foot movement, telescopic SLR is unable to meet task requirements in complex environments. In addition, the micro-robots in specific environments also apply the telescopic structures to greatly simplify the internal complexity and reduce mass and volume.
2. The articulated configuration exhibits distinct biomimetic features in jumping motion and is currently widely used in SLRs. This article classifies articulated SLR mechanisms into four (rigid, series elastic, parallel elastic, and variable stiffness based on actuators) and summarizes and analyzes the advantages and limitations of each type. Currently, improving motion efficiency and adaptability to complex environments and terrains through the application of elastic components has become a prevailing research trend. However, the inclusion of additional mechanisms increases the complexity and bulkiness of the overall structure, while also making controller design more challenging.
3. This article surveys various representative MLRs, systematically analyzing their key performance metrics and technical characteristics. It summarizes the challenges and issues encountered during the transition from SLRs to MLRs. These findings offer valuable insights for the future development and practical deployment of SLRs, supporting commercial applications and advancing critical enabling technologies.
4. This article classifies the current modeling methods into the SLIP model and the articulated model. Among them, the SLIP model has been widely applied and expanded due to its simplified structure and motion planning capabilities. However, the articulated model has gradually gained favor among researchers by adding elastic elements and dampers to more accurately capture the real dynamics of the prototype. The challenges faced by researchers in the modeling process include: energy loss caused by collisions with the ground, special structural effects on physical prototypes, and the complexity caused by additional linkage structures.
5. This paper places particular emphasis on the development trends of control strategies, elaborating on 5 model-based and 2 model-free control methods for categorization. It provides a comprehensive summary of the advantages and challenges associated with each method across different scenarios.

Further studies on biological inspiration, lightweight manufacture, auxiliary mechanisms, future materials, and intelligent control will contribute to enhancing dynamic performance, explosiveness, energy efficiency, environmental adaptability, and human–robot interaction.

## Data Availability

The data and materials included in this study are available from the corresponding author upon reasonable request.
